# Construction and testing of *Yarrowia lipolytica* recombinant protein expression chassis cells based on the high-throughput screening and secretome

**DOI:** 10.1186/s12934-023-02196-x

**Published:** 2023-09-15

**Authors:** Siqian Yu, Ge Zhang, Qi Liu, Yingping Zhuang, Zongjie Dai, Jianye Xia

**Affiliations:** 1 State Key Laboratory of Bioreactor Engineering, East China University of Science and Technology, Shanghai, 200237 China; 2 Key Laboratory of Engineering Biology for Low-Carbon Manufacturing, Tianjin Institute of Industrial Biotechnology, Chinese Academy of Sciences, Tianjin, 300308 China; 3 National Center of Technology Innovation for Synthetic Biology, Tianjin, 300308 China

**Keywords:** *Yarrowia lipolytica*, High-throughput screening, Secretome, Chassis cell

## Abstract

**Background:**

In the recombinant protein market with broad economic value, the rapid development of synthetic biology has made it necessary to construct an efficient exocrine expression system for the different heterologous proteins. *Yarrowia lipolytica* possesses unique advantages in nascent protein transport and glycosylation modification, so it can serve as a potential protein expression platform. Although the Po1 series derived from W29 is often used for the expression of the various heterologous proteins, the ability of W29 to secrete proteins has not been verified and the Po1 series has been found to be not convenient for further gene editing.

**Results:**

A total of 246 *Y. lipolytica* strains were evaluated for their secretory capacity through performing high-throughput screening in 48-well plate. Thereafter, following two rounds of shake flask re-screening, a high-secreting protein starting strain DBVPG 5851 was obtained. Subsequently, combined with the extracellular protein types and relative abundance information provided by the secretome of the starting strain, available chassis cell for heterologous protein expression were preliminarily constructed, and it was observed that the most potential signal peptide was derived from *YALI0D20680g*.

**Conclusions:**

This study offers a novel perspective on the diversification of *Y. lipolytica* host cells for the heterologous protein expression and provides significant basis for expanding the selection space of signal peptide tools in the future research.

**Supplementary Information:**

The online version contains supplementary material available at 10.1186/s12934-023-02196-x.

## Introduction

The importance of synthetic biology in the field of biomanufacturing cannot be ignored, where chassis cells serve as the key factors, providing both plasticity and controllability. Prior to this, *Escherichia coli* and *Saccharomyces cerevisiae* acting as chassis cells inspired unlimited creativity and potential in the field of biomanufacturing [1–3]. Thus, in this context, huge economic value contained in the recombinant protein market has triggered an urgent need for the construction of highly efficient chassis cells [4, 5]. In addition, recombinant proteins can be potentially divided into intracellular accumulation and secretory types due to their different localization. The latter, by directly secreting the different proteins into the medium, offers numerous advantages such as simplifying purification steps, improving product purity, and avoiding intracellular problems, thus making them highly useful for use [6].

*Yarrowia lipolytica* is an unconventional yeast that can efficiently secrete various proteins as well as organic acids. It has excellent physiological characteristics such as sufficient supply of acetyl-CoA and reducing power, so it has emerged as an important protein expression platform with immense potential in recent years. So far, more than 150 recombinant proteins have been expressed in *Y. lipolytica* [7, 8]. *Y. lipolytica* is basically identical to several other yeasts in the core pathway of protein secretion, but it also exhibits several unique metabolic characteristics, which could be the key to its efficient protein secretion. It has been found that secretome of *Y. lipolytica* was nearly twice that of *S. cerevisiae* (299 vs 156) [9], and the protein sequence in its secretory pathway was 40% more similar to that of mammals in comparison to that of budding yeast [10]. The complex secretion mechanism of *Y. lipolytica* could have laid the foundation for its ability to produce high-level secreted proteins. In addition, *Y. lipolytica* prefers to translocate the polypeptides from the ribosome to the endoplasmic reticulum in the form of co-translational transport (signal recognition particle-dependent) [11, 12], which can effectively avoid polypeptide aggregation in the cytoplasm and thereby save both energy and time.

*Y. lipolytica* provides strong support for the development and optimization of the various protein expression systems with its excellent protein secretion ability. At present, the Po1 series derived from W29 are commonly used to produce the different heterologous proteins [13], but its ability to secrete the proteins has not been compared with other wild strains on a large scale. It has been stablished that optimal selection of a high-secreting protein starting strain is crucial for the construction of chassis cells, as it may imply that the strain itself has stronger adaptability as well as competitive advantages. Moreover, limited application of the CRISPR-Cas9 system in the Po1 series, due to the lack of integration of Cas9 protein into its genome and the absence of *ku70* knockout has also hindered the precise editing and genetic modification of specific genes. In this study, we have evaluated the secretory capacity of 246 *Y. lipolytica* strains by using high-throughput screening in 48-well plates, and obtained a starting strain which exhibited better performance than W29. The study of the secretome developed for the starting strain provides novel clues both for the protease knockout strategy and the selection of novel signal peptides (SPs). Based on the screened starting strains and omics information, we have preliminarily constructed a chassis cell for the heterologous protein expression and identified the most potent SPs by evaluating the efficiency of different SPs to facilitate the secretion and expression of recombinant enzymes. The above work provides enormous possibilities and options for diversifying *Y. lipolytica* heterogeneous protein expression chassis cells and broadening the genetic tools such as SPs.

## Materials and methods

### Strains and routine culturing conditions

All the strains and plasmids used in this study have been listed in Additional file 1: Table S1. *Escherichia coli* strains were grown in Luria–Bertani (LB) medium (liquid or solidified with agar) supplemented with appropriate antibiotics when necessary according to standard protocols [14]. *Y. lipolytica* strains were cultured in yeast peptone dextrose (YPD) medium (liquid or solidified with agar) supplemented with appropriate antibiotics when necessary according to standard protocols [14]. The shake flask culture of *Y. lipolytica* strains with high-level secretory capacity was carried out in Delft medium (containing 7.5 g/L (NH_4_)_2_SO_4_, 14.4 g/L KH_2_PO_4_, 0.5 g/L Mg_2_SO_4_·7H_2_O, and 20.0 g/L C_6_H_12_O_6_, pH = 6.0) and PPB medium (containing 1.32 g/L yeast extraction, 1.32 g/L NH_4_Cl, 0.32 g/L KH_2_PO_4_, 0.13 g/L Mg_2_SO_4_, 0.33 mg/L thiamine, and 20.00 g/L C_6_H_12_O_6_) with shaking at 220 rpm. The preliminary screening in 48-well plates was performed in 1/4 Delft medium (containing 1.88 g/L (NH_4_)_2_SO_4_, 3.60 g/L KH_2_PO_4_, 0.5 g/L Mg_2_SO_4_·7H_2_O, and 5.00 g/L C_6_H_12_O_6_, pH = 6.0) with shaking at 800 rpm.

### Screening of high-secreting protein Y. lipolytica strains in 48-well plates and shake flasks

The various single clones picked from agar plates were cultured and preserved in 96-well plates, and then the Biomek i7 (Beckman Ltd., USA) automated workstation was used to complete the storage of multiple copies of the sample. In the MicroScreen system (Gering Ltd., China), the growth of *Y. lipolytica* strains was examined. We transferred 10 μL of the bacterial liquid preserved in the 96-well plate to a seed 48-well plate with 1 mL of 1/4 Delft medium in each well and cultured it for 24–36 h. Thereafter, starting with an initial *OD*_600_ of 0.2, an appropriate volume of bacterial liquid from the seed 48-well plate was transferred to a fermentation 48-well plate for further cultivation (each strain has 4 repetitions). Then the accumulation of extracellular total protein was determined by Bradford assay [15]. The preliminarily screened strains were subjected to re-screening in 250 mL shake flasks at 25 ℃.

### Sample preparation and detection of secretome

The supernatant samples obtained from the fermentation broth were desalted and concentrated using Amicon^®^ Ultra-4 Centrifugal Filter Unit (Millipore) with a 3 kDa molecular weight cutoff (MWCO) value. We first took an appropriate volume of the concentrated supernatant sample, mixed it with sodium dodecyl sulfate–polyacrylamide gel electrophoresis (SDS-PAGE), sample buffer and then completely denatured it at 95 °C. The separated protein components in the samples were then analyzed using 4–20% protein electrophoresis precast gels. The concentrated supernatant and the thick band sample separated by SDS-PAGE were thereafter subjected to trypsin digestion, followed by identification using LC–MS/MS.

Eksigent NanoLC-Ultra nanoliter liquid phase system was employed: 10 cm chromatographic column, inner diameter 75 μm, C18 filler, particle size 1.9 μm. Mobile phase A was 2% acetonitrile, 98% water (containing 0.2% formic acid), and mobile phase B was 98% acetonitrile, 2% water (containing 0.2% formic acid). The gradient separation was performed for 30 min and the mobile phase B was linearly increased from 5 to 40%. The SCIEX TripleTOF 5600 mass spectrometry system can perform information-dependent scanning acquisition data in positive ion mode. The WIFF files collected by the mass spectrometry were processed using the Paragon search engine of ProteinPilot v5.0 (AB SCIEX) software. The *Y. lipolytica* species database was downloaded from Uniprot [16] (https://www.uniprot.org/), which contained a total of 20,113 different protein sequences. The samples were aligned against this database and a false discovery rate (FDR) of 1% was set as the threshold for identifying the various significant matches.

### Prediction of secretory proteins and Gene Ontology functional enrichment analysis

The proteins can be released into the extracellular space through both the classical and non-classical secretory pathways [17]. Proteins belonging to the classical secretory pathway, which is characterized by the absence of transmembrane domains, lack of GPI anchor motif, and the presence of N-terminal SP sequences can be effectively predicted using software such as SignalP-5.0 [18], TMHMM [19], and Big-PI predictor. On the contrary, proteins belonging to the non-classical secretory pathway lack SP sequences detectable by SignalP-5.0 and have a Sec/P > 0.5 as predicted by SecretomeP 2.0 [20] (Fig. 2A). Therefore, further prediction and differentiation of the credible protein types, identified by mass spectrometry with a minimum of 10 matched peptide segments, can be performed to exclude intracellular protein release caused by the physiological cell lysis. GO functional enrichment analysis of the secretory proteins was conducted on the website (http://geneontology.org/).

### DNA manipulation techniques

The genomic DNA was extracted from *Y. lipolytica* using the Single-tube LiOAc-SDS lysis method [21]. The synthesis of the primers, codon-optimized *IPP1* sequences and DNA sequencing were all provided by Tsingke Biotech (Beijing, China). The sequences of the primers used in this study have been listed in Additional file 1: Table S2. Transformation of *Y. lipolytica* was performed according to the lithium acetate method [22]. The plasmid extraction was performed by using the AxyPrep Plasmid Miniprep Kit (Axygen, Hangzhou, China), PCR cleaning and gel extraction by using the AxyPrep DNA Gel Extraction Kit (Axygen, Hangzhou, China). The vector and target fragments were assembled using the ClonExpress II One Step Cloning Kit (Vazyme Biotech, Nanjing, China). Restriction endonuclease, *Asi*S I and *Not* I (NEB, Beijing, China) were used, following the manufacturer’s instructions.

### Knockout of ku70 and integration of Cas9

*Y. lipolytica* can display resistance to most antibiotics, with sensitivity observed only towards a few antibiotics such as Hygromycin B, Boromycin, and Nourseothricin [23]. Hence, to determine the appropriate selection pressure, the strains were evaluated for susceptibility to different concentrations of antibiotics (Additional file 1: Table S3).

The *ku70* was knocked out and Cas9 was integrated using the laboratory-stocked p4906-ku70-Cas9. Moreover, prior to this, the homologous arm sequences on the plasmid were validated for their consistency with the starting strain using Blastn [24]. Three distinct pairs of primers were designed to confirm the correct homologous recombination on the genome of the starting strain (Additional file 1: Fig. S1). Subsequently, the hygromycin B resistance gene (*hyg*^*R*^) was circularized and degraded using the Cre-LoxP system [25].

### Gene knockout and target protein integration based on CRISPR-Cas9

Based on CRISPR-Cas9, the complete coding sequence (CDS) of genes *AXP* and *XPR2*, encoding acid extracellular protease (AXP) and alkaline extracellular protease (AEP) respectively were removed to achieve optimal gene knockout. Amplify and ligation the promoter sequence (BB1635), N20 sequence, tracrRNA sequence, and terminator sequence (BB1636) required for constructing the guide RNA (gRNA) [26]. Subsequently, the obtained fragments were subjected to Gibson Assembly with pCfB3405 digested by *Asi*S I enzyme and the resulting reaction products were directly transformed into DH5α. The confirmation was conducted through Sanger sequencing, thus yielding a gRNA (pCfB3405-gAXP) targeting knockout site of *AXP.* Using pCfB3405-gAXP as a template, the N20 sequence was replaced to obtain a gRNA (pCfB3405-gXPR2) targeting knockout site of *XPR2*. The various repair templates for the knockout sites were generated by amplifying approximately 600 bp fragments upstream and downstream of the target sites, followed by their ligation. The respective gRNA and DNA repair templates were co-transformed into the starting strain, followed by the selection on YPD agar plates containing nourseothricin. The positive clones were further confirmed by using colony polymerase chain reaction (PCR) and Sanger sequencing.

Similarly, CRISPR-Cas9 was used to achieve the integration of the target proteins. Thus, by using pCfB3405-gAXP as a template, the N20 sequence targeting the protein integration site was replaced, thereby resulting in pCfB3405-gIntC_3. The protein expression cassettes were integrated as the repair templates and the fragments within the expression cassettes were connected using two rounds of SOE-PCR. Thereafter, following transformation into the chassis cells, the positive transformants were selected on YPD agar plates containing nourseothricin, and recombinant strains were obtained after the verification.

### Expression and activity determination of recombinase

The fermentation supernatants were collected and concentrated by using an Amicon^®^ Ultra-4 Centrifugal Filter Unit with a 10 kDa molecular weight cutoff (MWCO) value by centrifuging at 7500 × g and 4 ℃ to obtain the samples of extracellular proteins. In addition, intracellular protein samples were obtained by grinding at − 20 ℃, and an appropriate amount of protease inhibitor was added during the process. Afterwards, the recombinant inorganic pyrophosphatase (rPPase) expressed by the recombinant *Y. lipolytica* strain was analyzed by SDS-PAGE.

In this experiment, 1 unit (U) of enzyme activity was defined as the amount of enzyme required to generate 1 μmol of phosphate per minute under standard reaction conditions (50 mM Tris–HCl, pH 8.0, 25 °C). The enzymatic activity of rPPase was determined by employing an improved colorimetric method [27] and the required reagents have been listed in Additional file 1: Table S4. For the protein sample analysis, 500 μL of P buffer solution was first mixed with 50 μL of enzyme solution and preheated at 25 ℃. Subsequently, 20 μL of substrate was added to initiate the catalytic reaction, which was allowed to proceed for 30 min. The reaction was then terminated by adding 20 μL of 1 M citric acid. A mixture of 250 μL of the reaction solution and 2 mL of AAM solution was prepared at the room temperature and incubated for 3 min before measuring the absorbance at 420 nm using a spectrophotometer. The enzyme activity of rPPase was determined based on the standard curve of phosphate ion concentration (Additional file 1: Fig. S2).

## Results

### Screening of high-secreting protein Y. lipolytica strain

**Fig. 1:**
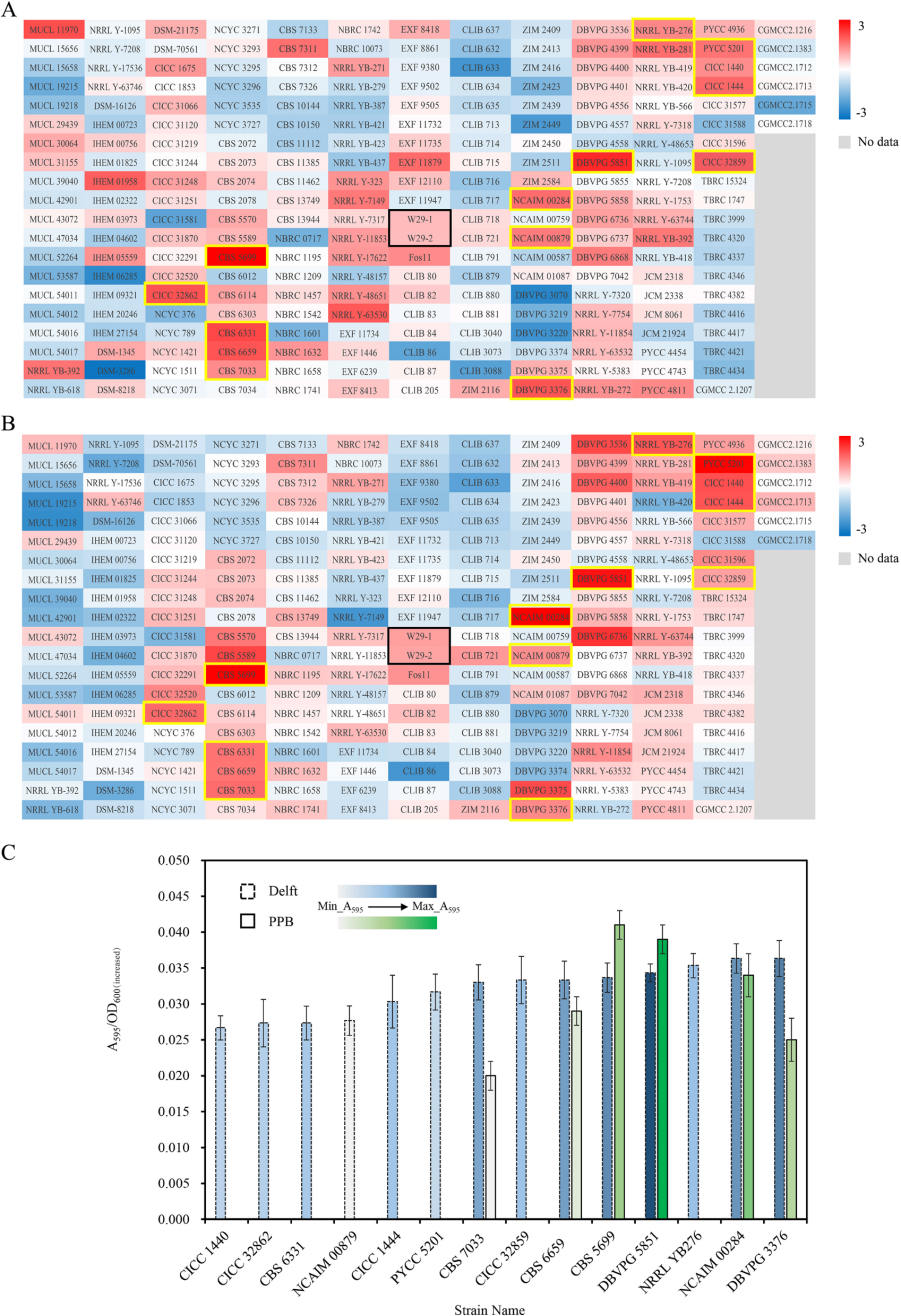
Screening of high-secreting protein *Y. lipolytica* strains. The relative accumulation of extracellular total protein (**A**) and relative accumulation of extracellular total protein produced by unit cell (**B**) of each strain during the preliminary screening in 48-well plates; (**C**) Shaking flask re-screening results based on Delft and PPB medium

During the high-throughput screening of the 48-well plate, Bradford assay [15] was used to determine the accumulation of extracellular total protein in each strain. The A_595_ is directly proportional to the actual protein concentration of each strain, so it was employed to directly characterize and compare the relative accumulation of extracellular total protein in each strain. In addition, the ratio of A_595_ to the increased *OD*_600_ of the strain (*OD*_600_ at the end of culture-initial *OD*_600_) denotes the relative accumulation of extracellular total protein produced by unit cell. The experimental data has been displayed in the form of heatmap after calculating the average and standardization with Z-Score (Fig. 1A, B).

The criteria for selecting the high-secreting protein strains encompass higher relative accumulation of extracellular total protein (A_595_) and accumulation of extracellular total protein produced by unit cell (A_595_/*OD*_600(increased)_). The color gradient, ranging from 3 to − 3, indicates a descending trend in the secretory capacity of the strains. Therefore, 14 strains highlighted within the yellow box were selected for further flask-shaking re-screening. Interestingly, CBS 5699 and DBVPG 5851 exhibited the highest relative accumulation of extracellular total protein with fold changes of 3.05 and 2.73 in comparison to W29, respectively. Moreover, the fold changes of CBS 5699, NCAIM 00284, DBVPG 5851, and PYCC 5201 with the highest relative accumulation of extracellular total protein produced by unit cell were 3.86, 3.85, 3.09 and 3.30 in comparison to W29, respectively (Additional file 1: Table S5).

Delft is a kind of inorganic salt medium with simple components, which can aid to preliminarily evaluate the enzyme-producing ability of the different *Y. lipolytica* strains (Fig. 1C, dashed bar). The ordinate is the extracellular total protein accumulation per unit cell and the depth of the column filling color denotes the relative accumulation of extracellular total protein at various levels. Based on the two screening criteria, CBS 6659, CBS 7033, DBVPG 5851, CBS 5699, NCAIM 00284 and DBVPG 3376 were selected for the next round of shake flask re-screening.

PPB is a medium commonly used in the production of different heterologous proteins in the fermentation industry [13], so the secretory capacity of the above six strains was examined in this medium in this round. The highest relative extracellular total protein accumulation as well as the second highest relative extracellular total protein accumulation per unit cell were observed in DBVPG 5851 (Fig. 1C, solid bar). It was thereafter speculated that the relatively high extracellular total protein accumulation per unit cell of CBS 5699 could be related to its lower biomass. Thus, after considering the results of two rounds of re-screening, DBVPG 5851, which appeared to perform better and remained stable, was ultimately selected as the starting strain with high-level of secretory capacity.

### Analysis of the secretome

**Fig. 2:**
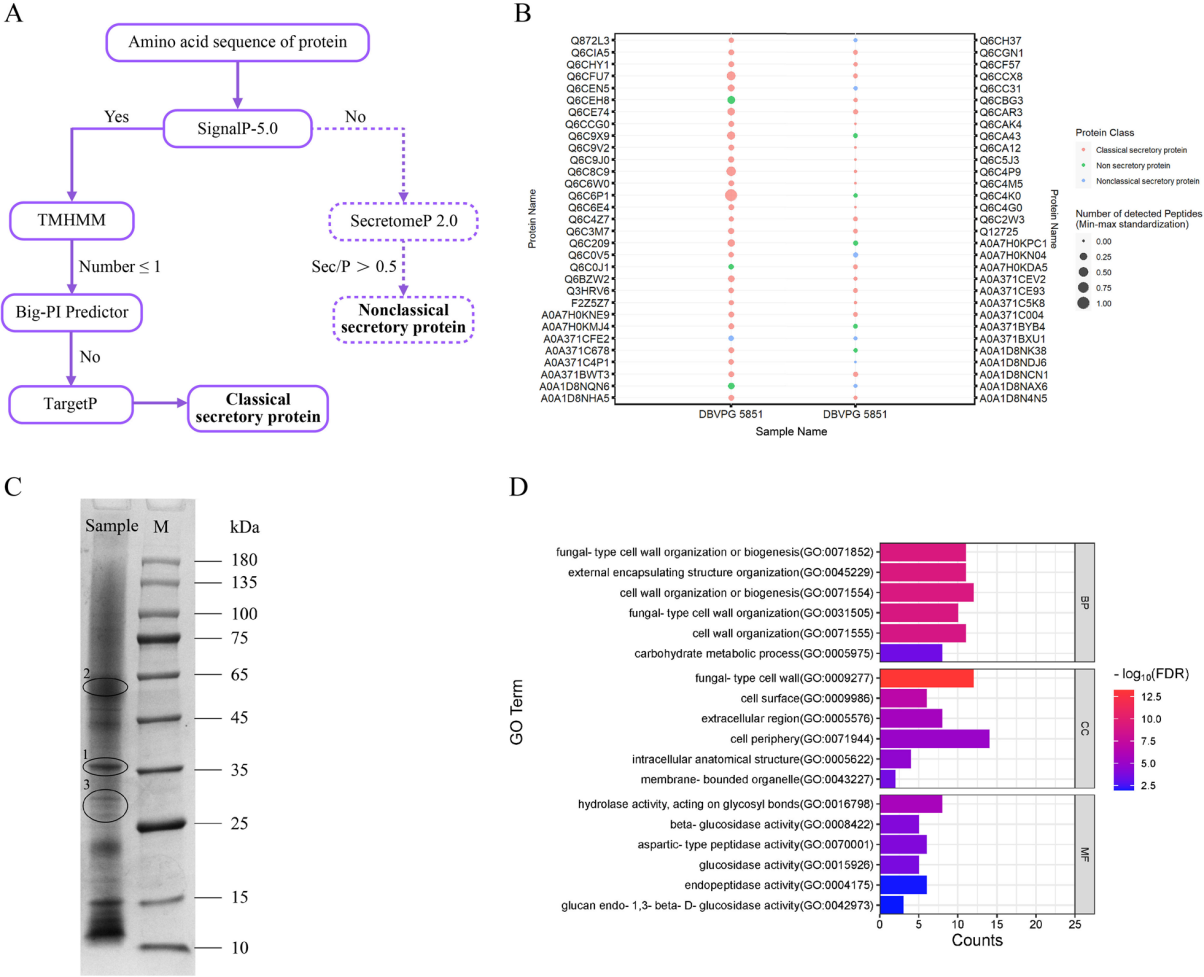
Secretome analysis of DBVPG 5851. **A** Flow chart for predicting the type of the secreted proteins; **B** Prediction results of secretome; **C** Protein gel electrophoresis profiles of DBVPG 5851; **D** Go functional enrichment of the secretory proteins

To understand the several types and relative abundance information of extracellular proteins in DBVPG 5851, a Label free secretome study was performed. Thus, after database matching analysis, a total of 408 proteins belonging to *Y. lipolytica* were identified, among which 62 credible proteins with peptide number ≥ 10 were matched. We then used tools such as SignalP to predict the types of the above 62 credible proteins (Fig. 2A) and the results have been represented by the bubble colors (Fig. 2B). The results indicated that there were 54 secretory proteins (47 classic secretory proteins and 7 non-classical secretory proteins), whereas the remaining 8 were considered to be the release of intracellular proteins caused by the physiological lysis of cells during the culture process.

The bubble size indicates the number of peptides matched to the corresponding protein (Fig. 2B). The larger the bubble, the more the number of peptides matched to the corresponding protein, and the higher the possibility of its existence. In addition, this parameter can also provide useful information about the relative abundance of a specific protein in the sample. Generally, proteins with higher abundance in the sample are more likely to produce a larger number of recognizable peptides [28]. Based on this, it was speculated that the proteins with higher extracellular abundance in DBVPG 5851 were Q6CFU7, Q6C8C9, and Q6C6P1, respectively.

To further verify the above speculation, three bands with good resolution and darker color (Fig. 2C) were cut for further identification. It was observed that the clearest and darkest band 1 was Q6CFU7, whereas Q6C8C9 was detected in the other two bands. This result was basically consistent with the above speculation, thus indicating that the number of the peptides matched to the protein can indeed explain the relative abundance of extracellular proteins in DBVPG 5851 to a certain extent. However, it was found that the molecular weight indicated by the band was different from the actual molecular weight of the identified protein. The presence of reducing agent and SDS in the SDS-PAGE sample buffer can effectively eliminate the influence of protein structure and charge on migration rate. As a result, glycosylation, a modification directly related to the chemical structure of the protein, was considered the primary cause underlying these findings [29]. Therefore, it was concluded that Q6CFU7, Q6C8C9, and Q6C6P1 exhibit higher abundance in the extracellular of DBVPG 5851. The higher abundance of these proteins can be attributed to their efficient SPs. Consequently, their secretion efficiency can be compared in the subsequent studies involving recombinant enzymes which can be guided by different SPs.

Moreover, to mitigate the reduction in the recombinant protein yield caused by excessive secretion of extracellular proteases, the presence of highly abundant proteases in the extracellular protein fraction of DBVPG 5851 was investigated through secretome. A total of 54 secreted proteins were subjected to GO functional enrichment analysis, thus revealing significant enrichment of glycoside hydrolase activity within the molecular function category (Fig. 2D). It was observed that only Q6C9J0 and Q6C6E4, which exhibited relatively low abundance, demonstrated significant protease activity. However, due to the limited availability of information such as enzyme activity, their knockout was not considered in the subsequent genetic modifications. Instead, the focus was placed on the knockout of *AXP* and *XPR2*, which have been extensively documented in the existing literature [13, 30, 31].

### Preliminary construction of chassis cell

**Fig. 3:**
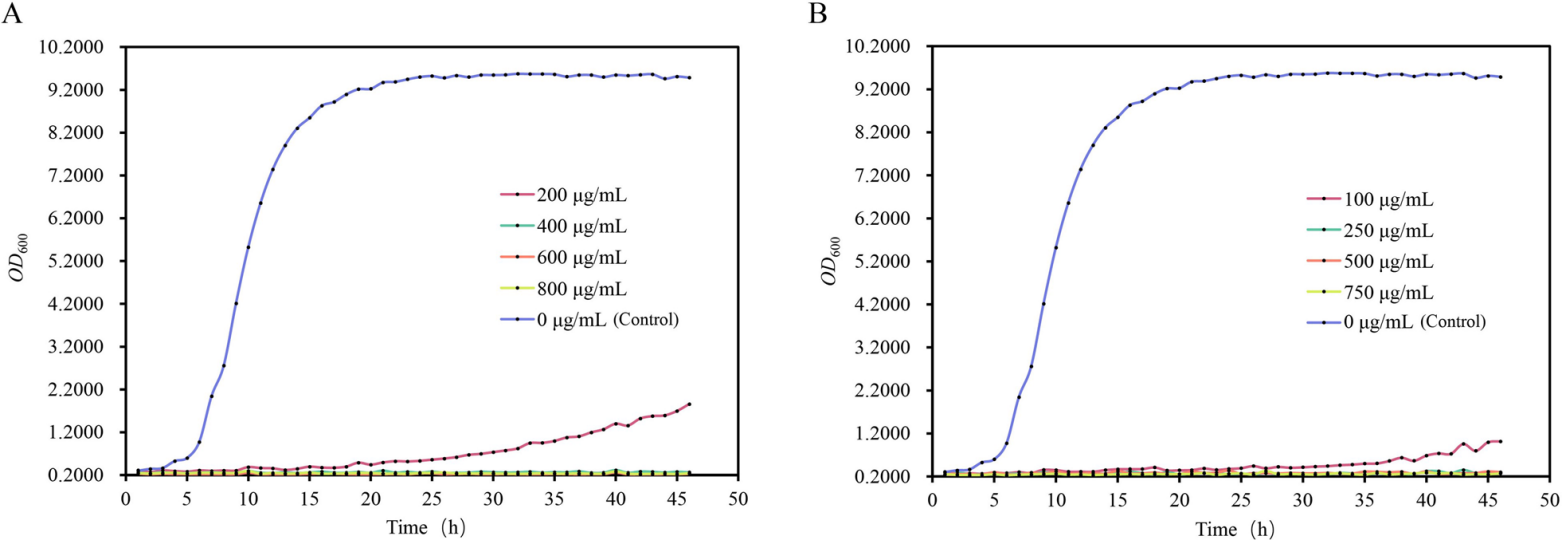
Potential effects of different concentrations of antibiotics on the growth of DBVPG 5851. **A** Effects of different concentrations of Hygromycin B on the growth of DBVPG 5851; **B** Effects of different concentrations of Nourseothricin on the growth of DBVPG 5851

The selection of appropriate antibiotic screening pressures can ensure a high success rate while at the same time minimize the loss of other valuable strains. The growth of DBVPG 5851 was found to be significantly inhibited when exposed to 200 μg/mL hygromycin B and 100 μg/mL nourseothricin, but varying degrees of growth were still observed with prolonged incubation. However, when the concentrations of hygromycin B and nourseothricin were increased to 400 μg/mL and 250 μg/mL and above, the growth of the strain was completely inhibited (Fig. 3). As a result, the screening of transformants was conducted at 400 μg/mL hygromycin B and 250 μg/mL nourseothricin.

To confirm the suitability of p4906-ku70-Cas9 (with homologous arms derived from W29) for direct transformation of DBVPG 5851, the sequence consistency between DBVPG 5851 and the corresponding locus in W29 was next evaluated using Blastn [24]. The nucleotide identities were found to be 100%, with no deletions or insertions of the base observed (Additional file 1: Table S6). This finding indicated a complete match between the two sequences, thereby affirming the direct applicability of p4906-ku70-Cas9 for transformation in DBVPG 5851.

To enhance the efficiency of the homologous recombination in DBVPG 5851 and ensure sustained as well as stable expression of the Cas9, we employed p4906-ku70-Cas9 for the knockout of *ku70* and integration of the Cas9 into its genome [32]. Subsequently, successful removal of the *hyg*^*R*^ was achieved using the Cre recombinase. The positive transformants were then subjected to consecutive subcultures to eliminate the plasmid, leading to the generation of a strain YYL 2572.

The presence of the high-level extracellular proteases can pose a significant threat to the production of recombinant proteins. Therefore, the genes *AXP* and *XPR2* encoding AXP and AEP were successively knocked out based on YYL 2572. This resulted in the creation of YYL 2573, with only *AXP* knocked out, and YYL 2574, a chassis strain with both *AXP* and *XPR2* knocked out. This practice can effectively serve to prevent the potential overexpression of AXP and AEP under intricate cultivation conditions.

### Construction of recombinant Y. lipolytica strains expressing rPPase

Holkenbrink and coworkers [26] in a previous study tested the expression intensity of the recombinant green fluorescent protein (GFP) under different integration sites and promoter guidance. They found that the integration site IntC_3 exhibited higher expression intensity and integration efficiency, whereas the promoter TEFin showed stronger expression levels. Therefore, IntC_3 and TEFin were selected as the integration site and promoter of the target protein, respectively. The tested protein was codon-optimized inorganic pyrophosphatase (*IPP1*) derived from *S. cerevisiae*. One of the SPs used was XPR2 pre, but the other three SPs were identified from the secretome with high abundance in the extracellular environment (Additional file 1: Table S7), namely Q6CFU7 (*YALI0B03564g*), Q6C8C9 (*YALI0D20680g*), and Q6C6P1 (*YALI0E07744p*).

**Fig. 4:**
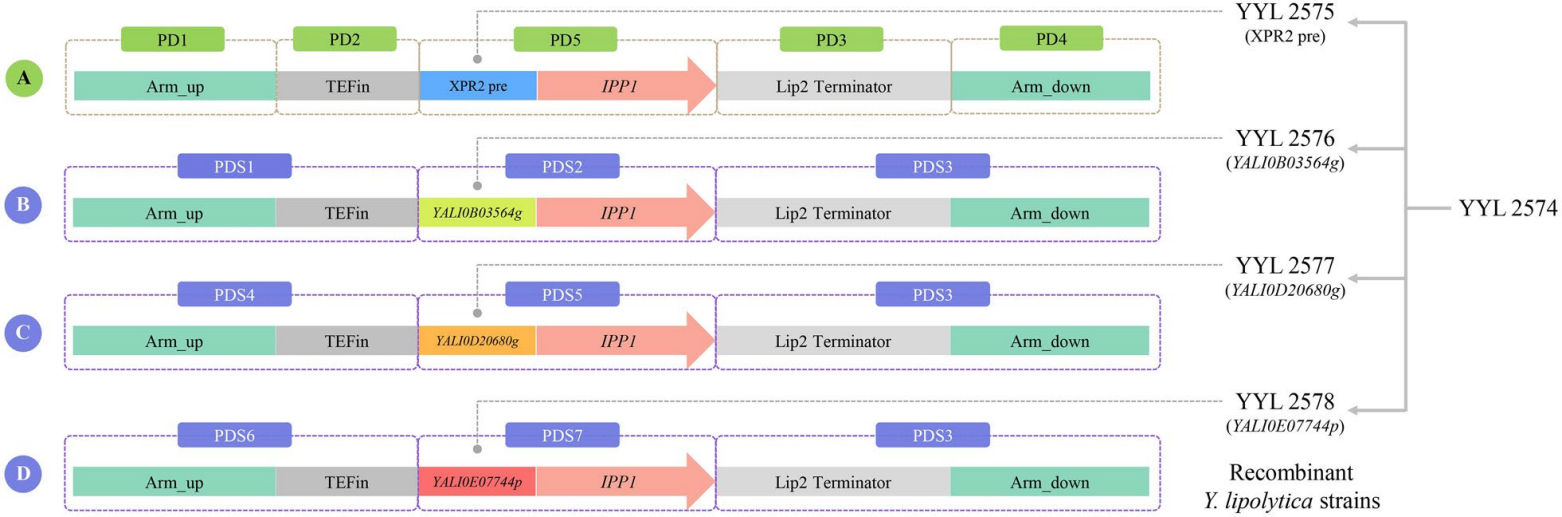
Construction of protein expression cassettes for expression of rPPase guided by different SPs and acquisition of recombinant *Y. lipolytica* strains

The protein expression cassette A was divided into five modules, which were connected and co-transformed with pCfB3405-gIntC_3 into YYL 2574, and strain YYL 2575 was obtained after the verification. The other three protein expression cassettes differed from cassette A only in the SPs and thus their construction was based on the cassette A as a template. Similarly, protein expression cassettes B, C, and D were separately co-transformed with pCfB3405-gIntC_3 into YYL 2574, and the strains YYL 2576, YYL 2577, and YYL 2578 were obtained after the verification (Fig. 4).

### Validation of chassis cell availability and evaluation of the signal peptide efficiency

**Fig. 5:**
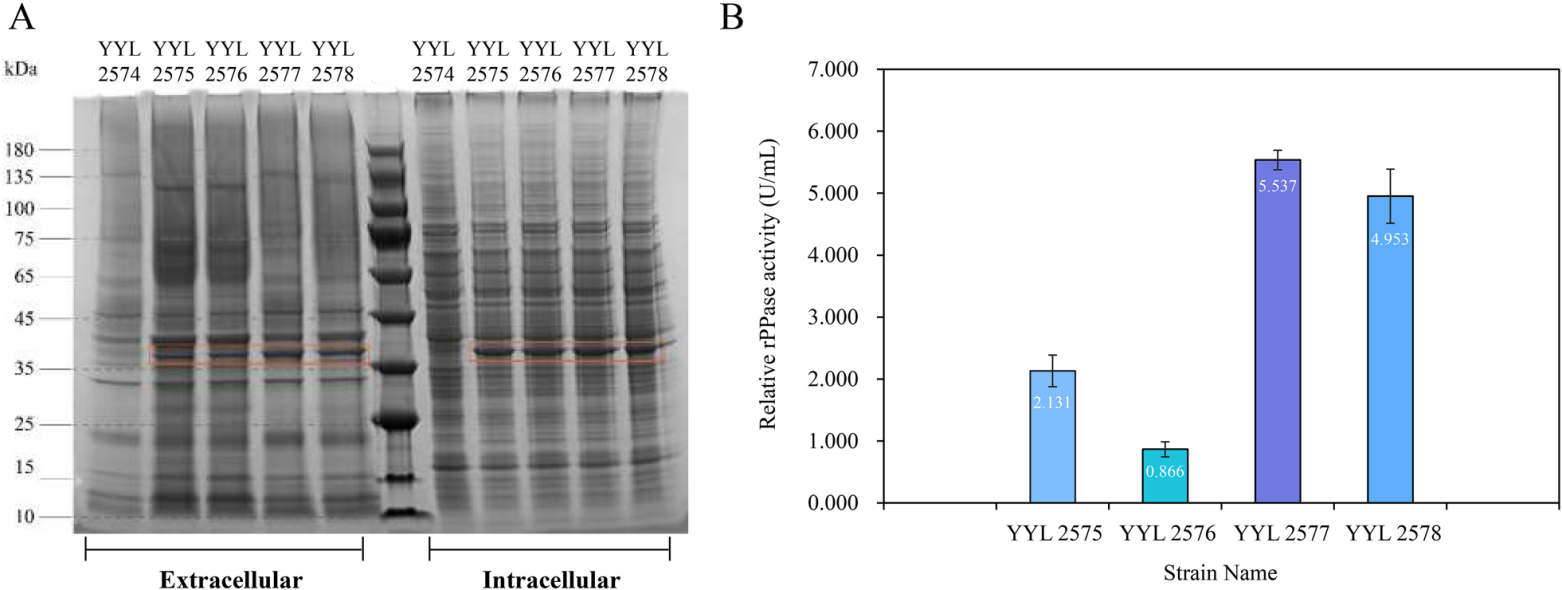
The expression of rPPase in intracellular and extracellular and determination of extracellular enzyme activity. **A** Detection of intracellular and extracellular rPPase expression using SDS-PAGE; **B** Determination of relative rPPase activity of recombinant *Y. lipolytica* strains

The expression of intracellular and extracellular rPPase was examined by SDS-PAGE. YYL 2574 was used as a control and the presence of the target protein bands was observed in the four additional strains that integrated the target gene (Fig. 5A). However, upon comparing the band intensities between both intracellular and extracellular samples, it was observed that rPPase appeared to be predominantly retained in the intracellular sample. Nevertheless, this observation provided evidence for possible application of the chassis cell at least.

In addition, the relative enzyme activity of the rPPase guided by the different SPs were analyzed (Additional file 1: Table S8). The results indicated that rPPase expressed by YYL 2577 exhibited the highest enzyme activity at 5.537 ± 0.159 U/mL, followed by YYL 2578 at 4.953 ± 0.438 U/mL (Fig. 5B). This finding probably indicated that SPs from *YALI0D20680g* and *YALI0E0744p* integrated into these strains possess significantly stronger guidance efficiency in comparison to the commonly used XPR2 pre. Remarkably, our findings align harmoniously with the observations made by Celinska [33], but we innovatively derived candidate SPs from experimental secretome and pioneeringly demonstrated their potential to guide the expression of rPPase.

## Discussion

The secretory recombinant protein expression system has emerged as the primary choice for producing a wide range of heterologous proteins due to its flexibility and high efficiency. Furthermore, yeast-based expression system, notably *Y. lipolytica*, have gained prominence as balanced alternatives to prokaryotic and mammalian counterparts. Müller [34] conducted a comparative analysis and identified *Y. lipolytica* as a promising replacement for *S. cerevisiae* due to its favorable attributes. Instead of directly comparing the expression levels of heterologous proteins in different strains that integrated the target protein [13, 35, 36], we conducted an extensive comparison of capacity to secrete homologous proteins in 246 *Y. lipolytica* strains. We believe that high-secreting protein strains often possess certain adaptability and competitive advantages, which may be reflected in more effective secretory pathways or mechanisms. And based on this, a strain DBVPG 5851 with superior performance was obtained. This approach avoids the tedious process of protein integration and construction, greatly saving time and resources, but its limitations need to be addressed after a comprehensive understanding of the distinctions in secretory mechanisms between homologous and heterologous proteins.

In this study, the secretome was carried out around the screened high-secreting protein strain DBVPG 5851 to explore the composition and relative abundance of its extracellular proteins. Interestingly, numerous prior studies have demonstrated that *Y. lipolytica* is capable of secreting high levels of AEP and AXP under the specific nutritional conditions [30, 31, 37]. Therefore, they are often knocked out in for the genetic modification of chassis cells, such as Po1f [13]. Building upon insights from the secretome and existing literatures, a protease knockout strategy for preferentially knocking out AEP and AXP was determined, and the innovative strategy offers a novel perspective for refining chassis cells.

High-efficiency SPs are essential for achieving high-yield production of the target proteins and preventing protein accumulation or mislocalization within the cells. In *Y. lipolytica*, SPs derived from *XPR2* and *LIP2* are widely used options [7], which are attached to the N-terminus of the newly synthesized polypeptide chains to direct the nascent proteins to their correct subcellular locations. Recently, Celinska [33] used genomic and transcriptomic data mining to identify and characterize novel signal peptides in *Y. lipolytica* and suggested a consensus sequence (MKFSAALLTAALA (S:V)AAAAA) of a potentially robust synthetic SP. Interestingly, the guiding performance results of SPs obtained by us through the tangible secretome experiments are basically consistent with the results of the Celinska’s research. This unique convergence of our experimental validation with the prior genomic exploration not only validates our findings but also emphasizes the broader significance of the identified SPs in mediating successful protein secretion.

Screening of dominant strains, construction of chassis cells, and optimization of secretory elements are pivotal for achieving high yields of recombinant proteins. To achieve targeted improvement and optimization in enzyme performance, it has become increasingly essential to finely control and guide the evolution of enzymes using directed evolution, enabling precise modifications of enzyme characteristics. The practical demand for efficient evaluation and screening of strains with improved activity or specificity has propelled the development of biosensors [38]. It uses the specific recognition capability of biomolecules to detect target molecules, and converts signals into readable output signals (such as fluorescent signals and cell growth rates), thereby enabling the detection and measurement of target molecules. This approach, which combines high sensitivity and specificity, has been extensively integrated with high-throughput screening techniques [39, 40]. By real-time monitoring of signals generated during enzyme-catalyzed reactions, timely feedback for directed evolution is provided, facilitating the expedited identification of high-performance strains.

## Conclusions

In this study, a high-secreting protein strain obtained by high-throughput screening and shake flask re-screening was used as the starting strain. It was then combined with the clues provided by secretome and a chassis cell for exocrine recombinant protein was preliminarily constructed. The successful expression of rPPase confirmed the availability of the chassis cell, and thereafter the secretion-guiding ability of three novel SPs based on the rPPase enzyme activity was examined and the data suggested that *YALI0D20680g* had the greater potential. This study provides novel perspectives on the diversity of chassis cells for heterologous protein expression in *Y. lipolytica* and expands the selection space for available SPs, thus offering broader possibilities for future research and applications.

### Supplementary Information

**Additional file 1: Table S1.** A list of various strains and plasmids used in this study. **Table S2.** A list of different primers used in this study. **Table S3.** Antibiotic concentration gradient. **Table S4.** Reagents required for the determination of rPPase activity. **Table S5.** Data summary table of preliminary high-throughput screening of various high-secreting protein strains in 48-well plate. **Table S6.** Comparison of the homology arm sequence on p4906-ku70-Cas9 with sequence on the corresponding locus of DBVPG 5851. **Table S7.** The sequences of the various signal peptides used in this study and their related information. **Table S8.** Relative activity of extracellular recombinant rPPase. **Fig S1.** Verification of PCR-based knockout of the ku70 and knock-in of the gene encoding the Cas9. **Fig S2.** Establishment of the phosphate standard curve.

## Data Availability

The authors promise the availability of supporting data.
