# Supplementary material for: Construction and testing of Yarrowia lipolytica recombinant protein expression chassis cells based on the high-throughput screening and secretome
Source: Microb Cell Fact. 2023 Sep 15;22:185. doi: 10.1186/s12934-023-02196-x (PMC10503192; doi:10.1186/s12934-023-02196-x)
Supplement: Supplementary file 1 — Additional file 1: Table S1. A list of various strains and plasmids used in this study. Table S2. A list of different primers used in this study. Table S3. Antibiotic concentration gradient. Table S4. Reagents required for the determination of rPPase activity. Table S5. Data summary table of preliminary high-throughput screening of various high-secreting protein strains in 48-well plate. Table S6. Comparison of the homology arm sequence on p4906-ku70-Cas9 with sequence on the corresponding locus of DBVPG 5851. Table S7. The sequences of the various signal peptides used in this study and their related information. Table S8. Relative activity of extracellular recombinant rPPase. Fig S1. Verification of PCR-based knockout of the ku70 and knock-in of the gene encoding the Cas9. Fig S2. Establishment of the phosphate standard curve. [file 12934_2023_2196_MOESM1_ESM.docx]

**Supporting Information**

**Supporting Table & Table legend**

**Table S1 A list of various** **strains and plasmids used in this study**

| Name | Description | Source |
| --- | --- | --- |
| Strains |  |  |
| *Escherichia coli* | | |
| DH5α | F-，lac ZΔM15 Δ，recA | Tsingke Bio. |
| *Yarrowia lipolytica* | | |
| DBVPG 5851 | WT | Lab stock |
| W29 | WT | Lab stock |
| YYL 2572 | Using DBVPG 5851 as the starting strain, the *ku70* was knocked out, and the gene encoding Cas9 was knocked in at this site (the selection marker hyg^R^ was recovered) | This study |
| YYL 2573 | Using YYL 2572 as the starting strain, the *AXP* was knocked out | This study |
| YYL 2574 | Using YYL 2573 as the starting strain, the *XPR2* was knocked out | This study |
| YYL 2575 | Using YYL 2574 as the starting strain, recombinant *Y. lipolytica* expressing *IPP1* guided by XPR2 pre | This study |
| YYL 2576 | Using YYL 2574 as the starting strain, recombinant *Y. lipolytica* expressing *IPP1* guided by *YALI0B03564g* (Q6CFU7) | This study |
| YYL 2577 | Using YYL 2574 as the starting strain, recombinant *Y. lipolytica* expressing *IPP1* guided by *YALI0D20680g* (Q6C8C9) | This study |
| YYL 2578 | Using YYL 2574 as the starting strain, recombinant *Y. lipolytica* expressing *IPP1* guided by *YALI0E07744p* (Q6C6P1) | This study |
| Plasmids | | |
| p4906-ku70-Cas9 | Integrated expression vector for knockout of *ku70* and knockin of gene encoding Cas9 | Lab stock |
| pYLCre | Remove the hyg^R^ resistance gene between two oriented LoxP sequences | Lab stock |
| pUC57-IPP1 | Contains codon-optimized *IPP1* sequence | Tsingke Bio. |
| pCfB3405 | Episomal vector backbone carrying the Nourseothricin resistance marker of *Y. lipolytica* for gRNA expression | Lab stock |
| pCfB3405-gAXP | gRNA plasmid targeting the *AXP* knockout site | This study |
| pCfB3405-gXPR2 | gRNA plasmid targeting the *XPR2* knockout site | This study |
| pCfB3405-gIntC_3 | gRNA plasmid targeting the IntC_3 integration site | This study |

**Table S2 A list of different** **primers used in this study.**

| Primer | Sequence (5’→3’) |
| --- | --- |
| ITS1 | TCCGTAGGTGAACCTGCGC |
| ITS4 | TCCTCCGCTTATTGATATGC |
| ku70up1 | CGTCTTCGCCTTCCTGTCTACT |
| ku70up2 | CCACGATGTTGCCGAAGATG |
| YSQ_01 | AAGAACCGAATCTGCTACCTGC |
| YSQ_02 | TGTCGTCCATCACAGTTTGCC |
| ku70dw1 | GCCGTGGTTGGCTTGTATG |
| ku70dw2 | TGTCCTAAATCACCGCTCCG |
| Cre_01 | GCTGCTCGGCATCACTATCAT |
| Cre_02 | GGCTTAGTGCCGAGTTTGGT |
| AXPxf_up1 | GAGTGAATTCAAGCGATTGTACGGC |
| AXPxf_up2 | GCTAGATAATAATAGTCTTTAGAATTGGAAGCTGATCTGGTGG |
| AXPxf_dw1 | CCAATTCTAAAGACTATTATTATCTAGCATTTCTTCTACAAGAAGCC |
| AXPxf_dw2 | GACATGTGTATTCTCCACACCGC |
| XPRxf_up1 | CTTGAGCGCCACGGTACATTCC |
| XPRxf_up2 | CTATCTGTTAATTGCCTGTTGGATTGGAGGATTGGATAGTGG |
| XPRxf_dw1 | CCAACAGGCAATTAACAGATAGTTTGCCGGTG |
| XPRxf_dw2 | CGGTTAAATCTCCGCCTCACTGC |
| AXP_yz1 | GAGCAGGGTTCCATCAAGTCG |
| AXP_yz2 | AGATGCGACGCCATTCCAG |
| XPR_yz1 | CAAGCCAACCATACAGCAGAAG |
| XPR_yz2 | AGTTGTTGGTCCTTCGGTCTG |
| sg10181 | ctaaatttgatgcaacggaatgcgtg |
| 2-gIntC_3 | CCCAAAGCCATAGCACTATTtaaccaacctgcgccgac |
| sg10182 | ttaaatttgcggccgcgaatg |
| 1-gIntC_3 | AATAGTGCTATGGCTTTGGGgttttagagctagaaatagcaagttaaaa taaggc |
| 2-gQ6C | TCGGACTCTCGCTTGACTCCtaaccaacctgcgccgac |
| 1-gQ6C | GGAGTCAAGCGAGAGTCCGAgttttagagctagaaatagcaagttaaaataaggc |
| 1-gRNA1121 | cgacttaacaacctcgccac |
| 2-gRNA1121 | gccaggcatcaaactaagcag |
| 1-Int_up | ACCACCATCCAATACCACGC |
| 2-ARMUP+PTEFS | cggtctcTACAGTGTCTATCAACGGGGCTT |
| 1-ARMUP+PTEFS | CCGTTGATAGACACTGTAgagaccgggttggcgg |
| 2-XPRpre+IPP1 | GTGTAGGTCATGGCCAGAACGGCAGTGAGAATAG |
| 1-XPRpre+IPP1 | CGTTCTGGCCATGACCTACACCACCCGACAG |
| 2-IPP1+TER | ccgaacagaagTTAGACGGAGCCGGAGATGAAGAAC |
| 1-IPP1+TER | GCTCCGTCTAActtctgttcggaatcaacctcaag |
| 2-TER+ARMDW | CTATGGCcagatgcattcttgggcgg |
| 1-TER+ARMDW | caagaatgcatctgGCCATAGCACTATTGTAGAGTGGC |
| PD4_dw1 | CGTAGACGCAGATTTGGCCG |
| 2-pro_FU7signal | CGGCAGCAAATGTGAACTTCATctgcggttagtactgcaaaaagtg |
| 1-FU7signal_IPP1 | ATGAAGTTCACATTTGCTGCCGTTACCGCCGCGCTGGCCTCGTCCGCCATGGCCATGACCTACACCACCCGACAG |
| 2-pro_8C9signal | AGGGCGGTGGAGAACTTCATctgcggttagtactgcaaaaagtg |
| 1-8C9signal_IPP1 | ATGAAGTTCTCCACCGCCCTTCTGGCTCTGGCCGCCGTCGCCACCGCCATGACCTACACCACCCGACAG |
| 2-pro_6P1signal | AGGCCAAACGAAAAGTGCATctgcggttagtactgcaaaaagtg |
| 1-6P1signal_IPP1 | ATGCACTTTTCGTTTGGCCTTCTGCTGGCCGCCTCTTCGGTTCTGGCTATGACCTACACCACCCGACAG |
| IntC3_yz1 | CAGATAGAGCAAGCGGGTGG |
| IntC3_yz2 | GGCTCCTGATGATTAGCGAAG |

**Table S3** **Antibiotic concentration gradient**

|  | 1 | 2 | 3 | 4 | 5 |
| --- | --- | --- | --- | --- | --- |
| Hygromycin B | 0 | 200 | 400 | 600 | 800 |
| Nourseothricin | 0 | 100 | 250 | 500 | 750 |

**Table S4** **Reagents required for the determination of rPPase activity**

|  | Reagent components |
| --- | --- |
| Reagent1（P Buffer） | 50 mM Tris-HCl (pH=8.0)，4 mg/mL MgSO_4_·7H_2_O，7.5 mg/mL KCl |
| Reagent 2 (Substrate) | 50 mM Na_4_P_2_O_7_ |
| Reagent 3*（AAM） | 2V Acetone, 1.25V 2 M H_2_SO_4_, 0.5V 20 mM (NH_4_)_6_Mo_7_O·4H_2_O, 0.25V H_2_O |

* AAM solution should be prepared and used immediately.

**Table S5 Data summary table of preliminary high-throughput screening of various high-secreting protein strains in 48-well plate**

1. **Relative accumulation of extracellular total protein (A_595_)**

| Strain name | Extracellular total protein_A_595_ | Extracellular total protein_A_595_ (Z-Score) | Fold changes (DBVPG 5851) | Fold changes (W29) |
| --- | --- | --- | --- | --- |
| MUCL 11970 | 0.8414 | 2.0747 | 1.0079 | 2.7049 |
| MUCL 15656 | 0.7366 | -0.2066 | / | / |
| MUCL 15658 | 0.6965 | -1.0794 | / | / |
| MUCL 19215 | 0.6662 | -1.7390 | / | / |
| MUCL 19218 | 0.6854 | -1.3221 | / | / |
| MUCL 29439 | 0.7705 | 0.5303 | 3.9433 | 0.6914 |
| MUCL 30064 | 0.7910 | 0.9781 | 2.1377 | 1.2753 |
| MUCL 31155 | 0.7780 | 0.6941 | 3.0127 | 0.9049 |
| MUCL 39040 | 0.7247 | -0.4656 | / | / |
| MUCL 42901 | 0.7198 | -0.5733 | / | / |
| MUCL 43072 | 0.7518 | 0.1238 | 16.8951 | 0.1614 |
| MUCL 47034 | 0.7326 | -0.2931 | / | / |
| MUCL 52264 | 0.7009 | -0.9831 | / | / |
| MUCL 53587 | 0.6725 | -1.6029 | / | / |
| MUCL 54011 | 0.7409 | -0.1135 | / | / |
| MUCL 54012 | 0.6948 | -1.1164 | / | / |
| MUCL 54016 | 0.7337 | -0.2691 | / | / |
| MUCL 54017 | 0.7120 | -0.7415 | / | / |
| NRRL YB-392 | 0.8216 | 1.6442 | 1.2717 | 2.1437 |
| NRRL YB-618 | 0.7088 | -0.8117 | / | / |
| NRRL Y-1095 | 0.7369 | -0.2006 | / | / |
| NRRL Y-7208 | 0.7281 | -0.3921 | / | / |
| NRRL Y-17536 | 0.7291 | -0.3698 | / | / |
| NRRL Y-63746 | 0.7540 | 0.1711 | 12.2204 | 0.2231 |
| DSM-16126 | 0.7097 | -0.7926 | / | / |
| IHEM 00723 | 0.7002 | -1.0000 | / | / |
| IHEM 00756 | 0.7705 | 0.5303 | 3.9433 | 0.6914 |
| IHEM 01825 | 0.7239 | -0.4830 | / | / |
| IHEM 01958 | 0.8329 | 1.8891 | 1.1069 | 2.4630 |
| IHEM 02322 | 0.7213 | -0.5396 | / | / |
| IHEM 03973 | 0.7672 | 0.4590 | 4.5557 | 0.5984 |
| IHEM 04602 | 0.6971 | -1.0675 | / | / |
| IHEM 05559 | 0.8036 | 1.2524 | 1.6696 | 1.6329 |
| IHEM 06285 | 0.6339 | -2.4414 | / | / |
| IHEM 09321 | 0.7163 | -0.6479 | / | / |
| IHEM 20246 | 0.7163 | -0.6479 | / | / |
| IHEM 27154 | 0.6972 | -1.0637 | / | / |
| DSM-1345 | 0.7914 | 0.9858 | 2.1212 | 1.2852 |
| DSM-3286 | 0.6147 | -2.8606 | / | / |
| DSM-8218 | 0.7709 | 0.5401 | 3.8717 | 0.7041 |
| DSM-21175 | 0.7886 | 0.9243 | 2.2623 | 1.2050 |
| DSM-70561 | 0.7366 | -0.2060 | / | / |
| CICC 1675 | 0.8020 | 1.2176 | 1.7173 | 1.5875 |
| CICC 1853 | 0.7426 | -0.0760 | / | / |
| CICC 31066 | 0.7840 | 0.8241 | 2.5372 | 1.0745 |
| CICC 31120 | 0.7570 | 0.2375 | 8.8043 | 0.3096 |
| CICC 31219 | 0.7534 | 0.1591 | 13.1397 | 0.2075 |
| CICC 31244 | 0.7420 | -0.0883 | / | / |
| CICC 31248 | 0.7957 | 1.0788 | 1.9382 | 1.4065 |
| CICC 31251 | 0.7627 | 0.3616 | 5.7831 | 0.4714 |
| CICC 31581 | 0.6580 | -1.9186 | / | / |
| CICC 31870 | 0.7766 | 0.6647 | 3.1458 | 0.8666 |
| CICC 32291 | 0.7505 | 0.0955 | 21.9031 | 0.1245 |
| CICC 32520 | 0.7793 | 0.7224 | 2.8946 | 0.9418 |
| CICC 32862 | 0.8393 | 2.0284 | 1.0309 | 2.6446 |
| NCYC 376 | 0.6783 | -1.4751 | / | / |
| NCYC 789 | 0.7188 | -0.5935 | / | / |
| NCYC 1421 | 0.7706 | 0.5335 | 3.9191 | 0.6956 |
| NCYC 1511 | 0.7446 | -0.0319 | / | / |
| NCYC 3071 | 0.7355 | -0.2310 | / | / |
| NCYC 3271 | 0.7391 | -0.1516 | / | / |
| NCYC 3293 | 0.7536 | 0.1635 | 12.7898 | 0.2132 |
| NCYC 3295 | 0.7235 | -0.4910 | / | / |
| NCYC 3296 | 0.6977 | -1.0528 | / | / |
| NCYC 3535 | 0.6993 | -1.0190 | / | / |
| NCYC 3727 | 0.7192 | -0.5848 | / | / |
| CBS 2072 | 0.7320 | -0.3072 | / | / |
| CBS 2073 | 0.7610 | 0.3251 | 6.4316 | 0.4239 |
| CBS 2074 | 0.7767 | 0.6669 | 3.1356 | 0.8694 |
| CBS 2078 | 0.7294 | -0.3627 | / | / |
| CBS 5570 | 0.7911 | 0.9790 | 2.1358 | 1.2765 |
| CBS 5589 | 0.7844 | 0.8334 | 2.5090 | 1.0866 |
| CBS 5699 | 0.8536 | 2.3397 | 0.8937 | 3.0505 |
| CBS 6012 | 0.7009 | -0.9831 | / | / |
| CBS 6114 | 0.7960 | 1.0857 | 1.9259 | 1.4155 |
| CBS 6303 | 0.7758 | 0.6456 | 3.2387 | 0.8418 |
| CBS 6331 | 0.8455 | 2.1628 | 0.9668 | 2.8199 |
| CBS 6659 | 0.8380 | 2.0012 | 1.0449 | 2.6091 |
| CBS 7033 | 0.8077 | 1.3411 | 1.5592 | 1.7485 |
| CBS 7034 | 0.7396 | -0.1423 | / | / |
| CBS 7133 | 0.7030 | -0.9374 | / | / |
| CBS 7311 | 0.8225 | 1.6638 | 1.2568 | 2.1692 |
| CBS 7312 | 0.7415 | -0.1004 | / | / |
| CBS 7326 | 0.7302 | -0.3459 | / | / |
| CBS 10144 | 0.7029 | -0.9401 | / | / |
| CBS 10150 | 0.6989 | -1.0277 | / | / |
| CBS 11112 | 0.6984 | -1.0392 | / | / |
| CBS 11385 | 0.7538 | 0.1668 | 12.5394 | 0.2174 |
| CBS 11462 | 0.7327 | -0.2925 | / | / |
| CBS 13749 | 0.7589 | 0.2794 | 7.4839 | 0.3643 |
| CBS 13944 | 0.7540 | 0.1711 | 12.2204 | 0.2231 |
| NBRC 0717 | 0.6703 | -1.6503 | / | / |
| NBRC 1195 | 0.7451 | -0.0226 | / | / |
| NBRC 1209 | 0.7402 | -0.1277 | / | / |
| NBRC 1457 | 0.7637 | 0.3826 | 5.4650 | 0.4988 |
| NBRC 1542 | 0.7476 | 0.0318 | 65.7633 | 0.0415 |
| NBRC 1601 | 0.6735 | -1.5808 | / | / |
| NBRC 1632 | 0.8025 | 1.2285 | 1.7021 | 1.6016 |
| NBRC 1658 | 0.7415 | -0.0994 | / | / |
| NBRC 1741 | 0.7466 | 0.0117 | 179.3166 | 0.0152 |
| NBRC 1742 | 0.7574 | 0.2468 | 8.4742 | 0.3217 |
| NBRC 10073 | 0.7210 | -0.5461 | / | / |
| NRRL YB-271 | 0.7992 | 1.1550 | 1.8104 | 1.5059 |
| NRRL YB-279 | 0.7130 | -0.7203 | / | / |
| NRRL YB-387 | 0.6947 | -1.1181 | / | / |
| NRRL YB-421 | 0.6981 | -1.0439 | / | / |
| NRRL YB-423 | 0.7186 | -0.5984 | / | / |
| NRRL YB-437 | 0.6910 | -1.1992 | / | / |
| NRRL Y-323 | 0.8413 | 2.0720 | 1.0092 | 2.7014 |
| NRRL Y-7149 | 0.8324 | 1.8793 | 1.1126 | 2.4502 |
| NRRL Y-7317 | 0.7668 | 0.4514 | 4.6326 | 0.5885 |
| NRRL Y-11853 | 0.7992 | 1.1566 | 1.8078 | 1.5080 |
| NRRL Y-17622 | 0.8014 | 1.2034 | 1.7375 | 1.5690 |
| NRRL Y-48157 | 0.6958 | -1.0958 | / | / |
| NRRL Y-48651 | 0.8063 | 1.3101 | 1.5961 | 1.7081 |
| NRRL Y-63530 | 0.8356 | 1.9473 | 1.0738 | 2.5389 |
| EXF 11734 | 0.7283 | -0.3867 | / | / |
| EXF 1446 | 0.7646 | 0.4035 | 5.1825 | 0.5260 |
| EXF 6239 | 0.7361 | -0.2185 | / | / |
| EXF 8413 | 0.7768 | 0.6674 | 3.1330 | 0.8702 |
| EXF 8418 | 0.7885 | 0.9226 | 2.2663 | 1.2029 |
| EXF 8861 | 0.7268 | -0.4199 | / | / |
| EXF 9380 | 0.7315 | -0.3176 | / | / |
| EXF 9502 | 0.7313 | -0.3225 | / | / |
| EXF 9505 | 0.7533 | 0.1575 | 13.2759 | 0.2053 |
| EXF 11732 | 0.7465 | 0.0089 | 233.8924 | 0.0117 |
| EXF 11735 | 0.7705 | 0.5314 | 3.9352 | 0.6928 |
| EXF 11879 | 0.8254 | 1.7259 | 1.2116 | 2.2501 |
| EXF 12110 | 0.7789 | 0.7148 | 2.9255 | 0.9319 |
| EXF 11947 | 0.7265 | -0.4264 | / | / |
| W29-1 | 0.7816 | 0.7719 | 2.7089 | 1.0064 |
| W29-2 | 0.7811 | 0.7621 | 2.7438 | 0.9936 |
| Fos11 | 0.8080 | 1.3476 | 1.5516 | 1.7570 |
| CLIB 80 | 0.7582 | 0.2625 | 7.9648 | 0.3423 |
| CLIB 82 | 0.7733 | 0.5923 | 3.5303 | 0.7722 |
| CLIB 83 | 0.7384 | -0.1674 | / | / |
| CLIB 84 | 0.7551 | 0.1951 | 10.7202 | 0.2543 |
| CLIB 86 | 0.6596 | -1.8832 | / | / |
| CLIB 87 | 0.7514 | 0.1145 | 18.2600 | 0.1493 |
| CLIB 205 | 0.7470 | 0.0198 | 105.4795 | 0.0258 |
| CLIB 637 | 0.7023 | -0.9532 | / | / |
| CLIB 632 | 0.6859 | -1.3107 | / | / |
| CLIB 633 | 0.6448 | -2.2059 | / | / |
| CLIB 634 | 0.7212 | -0.5418 | / | / |
| CLIB 635 | 0.7092 | -0.8041 | / | / |
| CLIB 713 | 0.7283 | -0.3883 | / | / |
| CLIB 714 | 0.7242 | -0.4770 | / | / |
| CLIB 715 | 0.7522 | 0.1325 | 15.7846 | 0.1727 |
| CLIB 716 | 0.6903 | -1.2144 | / | / |
| CLIB 717 | 0.7017 | -0.9668 | / | / |
| CLIB 718 | 0.7637 | 0.3833 | 5.4547 | 0.4998 |
| CLIB 721 | 0.7659 | 0.4301 | 4.8612 | 0.5608 |
| CLIB 791 | 0.7351 | -0.2392 | / | / |
| CLIB 879 | 0.6992 | -1.0201 | / | / |
| CLIB 880 | 0.7341 | -0.2621 | / | / |
| CLIB 881 | 0.7467 | 0.0138 | 151.1090 | 0.0180 |
| CLIB 3040 | 0.7337 | -0.2702 | / | / |
| CLIB 3073 | 0.7244 | -0.4716 | / | / |
| CLIB 3088 | 0.6552 | -1.9779 | / | / |
| ZIM 2116 | 0.7898 | 0.9509 | 2.1989 | 1.2398 |
| ZIM 2409 | 0.7189 | -0.5924 | / | / |
| ZIM 2413 | 0.7255 | -0.4493 | / | / |
| ZIM 2416 | 0.7126 | -0.7284 | / | / |
| ZIM 2423 | 0.6747 | -1.5540 | / | / |
| ZIM 2439 | 0.7142 | -0.6952 | / | / |
| ZIM 2449 | 0.6479 | -2.1373 | / | / |
| ZIM 2450 | 0.7473 | 0.0269 | 77.7376 | 0.0351 |
| ZIM 2511 | 0.6999 | -1.0054 | / | / |
| ZIM 2584 | 0.7827 | 0.7964 | 2.6256 | 1.0383 |
| NCAIM 00284 | 0.8252 | 1.7210 | 1.2150 | 2.2437 |
| NCAIM 00759 | 0.7333 | -0.2784 | / | / |
| NCAIM 00879 | 0.8169 | 1.5419 | 1.3561 | 2.0103 |
| NCAIM 00587 | 0.7050 | -0.8955 | / | / |
| NCAIM 01087 | 0.7536 | 0.1635 | 12.7898 | 0.2132 |
| DBVPG 3070 | 0.6296 | -2.5357 | / | / |
| DBVPG 3219 | 0.6703 | -1.6503 | / | / |
| DBVPG 3220 | 0.6417 | -2.2728 | / | / |
| DBVPG 3374 | 0.7151 | -0.6740 | / | / |
| DBVPG 3375 | 0.7886 | 0.9248 | 2.2610 | 1.2057 |
| DBVPG 3376 | 0.8374 | 1.9883 | 1.0516 | 2.5923 |
| DBVPG 3536 | 0.7786 | 0.7082 | 2.9525 | 0.9234 |
| DBVPG 4399 | 0.8024 | 1.2257 | 1.7059 | 1.5981 |
| DBVPG 4400 | 0.7761 | 0.6527 | 3.2036 | 0.8510 |
| DBVPG 4401 | 0.7637 | 0.3828 | 5.4624 | 0.4991 |
| DBVPG 4556 | 0.7628 | 0.3632 | 5.7571 | 0.4735 |
| DBVPG 4557 | 0.7253 | -0.4525 | / | / |
| DBVPG 4558 | 0.7133 | -0.7143 | / | / |
| DBVPG 5851 | 0.8422 | 2.0910 | 1.0000 | 2.7262 |
| DBVPG 5855 | 0.7395 | -0.1429 | / | / |
| DBVPG 5858 | 0.7910 | 0.9781 | 2.1377 | 1.2753 |
| DBVPG 6736 | 0.7971 | 1.1104 | 1.8831 | 1.4477 |
| DBVPG 6737 | 0.7724 | 0.5720 | 3.6556 | 0.7458 |
| DBVPG 6868 | 0.8162 | 1.5267 | 1.3696 | 1.9905 |
| DBVPG 7042 | 0.7487 | 0.0563 | 37.1507 | 0.0734 |
| NRRL Y-7320 | 0.7291 | -0.3698 | / | / |
| NRRL Y-7754 | 0.7816 | 0.7719 | 2.7089 | 1.0064 |
| NRRL Y-11854 | 0.7829 | 0.8013 | 2.6096 | 1.0447 |
| NRRL Y-63532 | 0.7807 | 0.7528 | 2.7775 | 0.9815 |
| NRRL Y-5383 | 0.7499 | 0.0819 | 25.5433 | 0.1067 |
| NRRL YB-272 | 0.8054 | 1.2916 | 1.6189 | 1.6840 |
| NRRL YB-276 | 0.8106 | 1.4035 | 1.4898 | 1.8299 |
| NRRL YB-281 | 0.8246 | 1.7095 | 1.2231 | 2.2288 |
| NRRL YB-419 | 0.7635 | 0.3790 | 5.5173 | 0.4941 |
| NRRL YB-420 | 0.7571 | 0.2391 | 8.7442 | 0.3118 |
| NRRL YB-566 | 0.7422 | -0.0841 | / | / |
| NRRL Y-7318 | 0.7655 | 0.4225 | 4.9489 | 0.5509 |
| NRRL Y-48653 | 0.7078 | -0.8346 | / | / |
| NRRL Y-1095 | 0.7246 | -0.4672 | / | / |
| NRRL Y-7208 | 0.7436 | -0.0547 | / | / |
| NRRL Y-17534 | 0.7626 | 0.3594 | 5.8181 | 0.4686 |
| NRRL Y-63744 | 0.7874 | 0.8992 | 2.3253 | 1.1724 |
| NRRL YB-392 | 0.8183 | 1.5724 | 1.3298 | 2.0501 |
| NRRL YB-418 | 0.7256 | -0.4465 | / | / |
| JCM 2318 | 0.7611 | 0.3262 | 6.4102 | 0.4253 |
| JCM 2338 | 0.7468 | 0.0155 | 135.1626 | 0.0202 |
| JCM 8061 | 0.7609 | 0.3224 | 6.4859 | 0.4203 |
| JCM 21924 | 0.7022 | -0.9554 | / | / |
| PYCC 4454 | 0.7274 | -0.4068 | / | / |
| PYCC 4743 | 0.7471 | 0.0220 | 95.0433 | 0.0287 |
| PYCC 4811 | 0.8093 | 1.3765 | 1.5191 | 1.7946 |
| PYCC 4936 | 0.7879 | 0.9107 | 2.2961 | 1.1873 |
| PYCC 5201 | 0.8211 | 1.6317 | 1.2815 | 2.1274 |
| CICC 1440 | 0.8069 | 1.3237 | 1.5797 | 1.7258 |
| CICC1444 | 0.8270 | 1.7601 | 1.1880 | 2.2948 |
| CICC 31577 | 0.7538 | 0.1684 | 12.4178 | 0.2195 |
| CICC 31588 | 0.6807 | -1.4228 | / | / |
| CICC 31596 | 0.7631 | 0.3697 | 5.6554 | 0.4821 |
| CICC 32859 | 0.8171 | 1.5457 | 1.3528 | 2.0153 |
| TBRC 15324 | 0.7380 | -0.1772 | / | / |
| TBRC 1747 | 0.7488 | 0.0595 | 35.1137 | 0.0776 |
| TBRC 3999 | 0.7154 | -0.6675 | / | / |
| TBRC 4320 | 0.7066 | -0.8601 | / | / |
| TBRC 4337 | 0.7036 | -0.9243 | / | / |
| TBRC 4346 | 0.7167 | -0.6392 | / | / |
| TBRC 4382 | 0.7470 | 0.0187 | 111.6071 | 0.0244 |
| TBRC 4416 | 0.7023 | -0.9532 | / | / |
| TBRC 4417 | 0.7212 | -0.5418 | / | / |
| TBRC 4421 | 0.6879 | -1.2666 | / | / |
| TBRC 4434 | 0.6806 | -1.4266 | / | / |
| CGMCC2.1207 | 0.7287 | -0.3791 | / | / |
| CGMCC2.1216 | 0.7614 | 0.3333 | 6.2741 | 0.4345 |
| CGMCC2.1383 | 0.7215 | -0.5363 | / | / |
| CGMCC2.1712 | 0.7398 | -0.1369 | / | / |
| CGMCC2.1713 | 0.7488 | 0.0590 | 35.4375 | 0.0769 |
| CGMCC2.1715 | 0.6630 | -1.8081 | / | / |
| CGMCC2.1718 | 0.7424 | -0.0803 | / | / |

1. **Relative accumulation of extracellular total proteins produced by unit cell (A_595_/*OD*_600 (increased)_)**

| Strain name | *OD*_600 (increased)_* | Extracellular total protein_ A_595_/*OD*_600 (increased)_ | Extracellular total protein_A_595_ (Z-Score) | Fold changes  (DBVPG 5851) | Fold changes (W29) |
| --- | --- | --- | --- | --- | --- |
| MUCL 11970 | 5.5036 | 0.1529 | 0.8066 | 3.4440 | 0.8965 |
| MUCL 15656 | 5.6913 | 0.1294 | -0.5115 | / | / |
| MUCL 15658 | 6.2137 | 0.1121 | -1.4856 | / | / |
| MUCL 19215 | 6.9168 | 0.0963 | -2.3721 | / | / |
| MUCL 19218 | 7.1870 | 0.0954 | -2.4259 | / | / |
| MUCL 29439 | 5.1821 | 0.1487 | 0.5703 | 4.8711 | 0.6338 |
| MUCL 30064 | 6.3273 | 0.1250 | -0.7592 | / | / |
| MUCL 31155 | 5.8482 | 0.1330 | -0.3091 | / | / |
| MUCL 39040 | 6.6155 | 0.1095 | -1.6287 | / | / |
| MUCL 42901 | 6.4521 | 0.1116 | -1.5159 | / | / |
| MUCL 43072 | 5.0821 | 0.1479 | 0.5281 | 5.2609 | 0.5869 |
| MUCL 47034 | 5.6029 | 0.1308 | -0.4366 | / | / |
| MUCL 52264 | 5.5319 | 0.1267 | -0.6644 | / | / |
| MUCL 53587 | 5.7879 | 0.1162 | -1.2557 | / | / |
| MUCL 54011 | 5.0541 | 0.1466 | 0.4530 | 6.1325 | 0.5035 |
| MUCL 54012 | 5.0747 | 0.1369 | -0.0907 | / | / |
| MUCL 54016 | 6.4547 | 0.1137 | -1.3967 | / | / |
| MUCL 54017 | 5.7609 | 0.1236 | -0.8391 | / | / |
| NRRL YB-392 | 6.1466 | 0.1337 | -0.2729 | / | / |
| NRRL YB-618 | 6.3911 | 0.1109 | -1.5523 | / | / |
| NRRL Y-1095 | 6.0499 | 0.1218 | -0.9401 | / | / |
| NRRL Y-7208 | 6.8766 | 0.1059 | -1.8348 | / | / |
| NRRL Y-17536 | 5.3108 | 0.1373 | -0.0698 | / | / |
| NRRL Y-63746 | 4.9498 | 0.1523 | 0.7750 | 3.5847 | 0.8613 |
| DSM-16126 | 6.4050 | 0.1108 | -1.5581 | / | / |
| IHEM 00723 | 5.2872 | 0.1324 | -0.3430 | / | / |
| IHEM 00756 | 6.1004 | 0.1263 | -0.6874 | / | / |
| IHEM 01825 | 6.2408 | 0.1160 | -1.2662 | / | / |
| IHEM 01958 | 6.2225 | 0.1338 | -0.2629 | / | / |
| IHEM 02322 | 6.2489 | 0.1154 | -1.2981 | / | / |
| IHEM 03973 | 6.1866 | 0.1240 | -0.8161 | / | / |
| IHEM 04602 | 6.3990 | 0.1089 | -1.6632 | / | / |
| IHEM 05559 | 6.3449 | 0.1267 | -0.6671 | / | / |
| IHEM 06285 | 5.6342 | 0.1125 | -1.4618 | / | / |
| IHEM 09321 | 5.0174 | 0.1428 | 0.2384 | 11.6546 | 0.2649 |
| IHEM 20246 | 5.4459 | 0.1315 | -0.3929 | / | / |
| IHEM 27154 | 5.1359 | 0.1358 | -0.1557 | / | / |
| DSM-1345 | 5.8256 | 0.1358 | -0.1508 | / | / |
| DSM-3286 | 5.7186 | 0.1075 | -1.7443 | / | / |
| DSM-8218 | 6.1007 | 0.1264 | -0.6836 | / | / |
| DSM-21175 | 6.1181 | 0.1289 | -0.5417 | / | / |
| DSM-70561 | 5.9936 | 0.1229 | -0.8781 | / | / |
| CICC 1675 | 6.2689 | 0.1279 | -0.5952 | / | / |
| CICC 1853 | 6.1477 | 0.1208 | -0.9966 | / | / |
| CICC 31066 | 5.8393 | 0.1343 | -0.2402 | / | / |
| CICC 31120 | 5.4235 | 0.1396 | 0.0590 | 47.0493 | 0.0656 |
| CICC 31219 | 5.4456 | 0.1384 | -0.0099 | / | / |
| CICC 31244 | 4.9520 | 0.1498 | 0.6360 | 4.3680 | 0.7068 |
| CICC 31248 | 5.4508 | 0.1460 | 0.4182 | 6.6428 | 0.4648 |
| CICC 31251 | 4.9768 | 0.1533 | 0.8275 | 3.3574 | 0.9196 |
| CICC 31581 | 5.9160 | 0.1112 | -1.5348 | / | / |
| CICC 31870 | 5.2211 | 0.1487 | 0.5743 | 4.8373 | 0.6383 |
| CICC 32291 | 4.9259 | 0.1524 | 0.7769 | 3.5758 | 0.8634 |
| CICC 32520 | 4.7560 | 0.1639 | 1.4231 | 1.9521 | 1.5816 |
| CICC 32862 | 4.9348 | 0.1701 | 1.7727 | 1.5672 | 1.9700 |
| NCYC 376 | 4.9219 | 0.1378 | -0.0398 | / | / |
| NCYC 789 | 5.6669 | 0.1268 | -0.6564 | / | / |
| NCYC 1421 | 5.4249 | 0.1420 | 0.1979 | 14.0383 | 0.2199 |
| NCYC 1511 | 5.6314 | 0.1322 | -0.3540 | / | / |
| NCYC 3071 | 5.6643 | 0.1298 | -0.4881 | / | / |
| NCYC 3271 | 5.9461 | 0.1243 | -0.7993 | / | / |
| NCYC 3293 | 5.4340 | 0.1387 | 0.0087 | 320.4953 | 0.0096 |
| NCYC 3295 | 5.3960 | 0.1341 | -0.2496 | / | / |
| NCYC 3296 | 5.5381 | 0.1260 | -0.7048 | / | / |
| NCYC 3535 | 5.7317 | 0.1220 | -0.9287 | / | / |
| NCYC 3727 | 5.8150 | 0.1237 | -0.8342 | / | / |
| CBS 2072 | 4.7750 | 0.1533 | 0.8298 | 3.3480 | 0.9222 |
| CBS 2073 | 5.0548 | 0.1506 | 0.6760 | 4.1099 | 0.7512 |
| CBS 2074 | 5.1065 | 0.1521 | 0.7629 | 3.6414 | 0.8479 |
| CBS 2078 | 5.4158 | 0.1347 | -0.2159 | / | / |
| CBS 5570 | 4.7386 | 0.1669 | 1.5966 | 1.7400 | 1.7744 |
| CBS 5589 | 4.6260 | 0.1696 | 1.7438 | 1.5931 | 1.9380 |
| CBS 5699 | 4.2623 | 0.2003 | 3.4692 | 0.8008 | 3.8556 |
| CBS 6012 | 5.5181 | 0.1270 | -0.6465 | / | / |
| CBS 6114 | 5.3579 | 0.1486 | 0.5638 | 4.9273 | 0.6266 |
| CBS 6303 | 5.1167 | 0.1516 | 0.7352 | 3.7787 | 0.8171 |
| CBS 6331 | 5.0389 | 0.1678 | 1.6442 | 1.6896 | 1.8273 |
| CBS 6659 | 5.0110 | 0.1672 | 1.6134 | 1.7219 | 1.7931 |
| CBS 7033 | 4.7715 | 0.1693 | 1.7279 | 1.6077 | 1.9204 |
| CBS 7034 | 5.1220 | 0.1444 | 0.3293 | 8.4369 | 0.3659 |
| CBS 7133 | 5.4613 | 0.1287 | -0.5506 | / | / |
| CBS 7311 | 5.0620 | 0.1625 | 1.3466 | 2.0630 | 1.4966 |
| CBS 7312 | 4.9805 | 0.1489 | 0.5815 | 4.7773 | 0.6463 |
| CBS 7326 | 4.8312 | 0.1511 | 0.7089 | 3.9191 | 0.7878 |
| CBS 10144 | 5.0891 | 0.1381 | -0.0230 | / | / |
| CBS 10150 | 5.4888 | 0.1273 | -0.6294 | / | / |
| CBS 11112 | 5.3984 | 0.1294 | -0.5151 | / | / |
| CBS 11385 | 5.3746 | 0.1402 | 0.0964 | 28.8231 | 0.1071 |
| CBS 11462 | 5.4196 | 0.1352 | -0.1878 | / | / |
| CBS 13749 | 4.8856 | 0.1553 | 0.9447 | 2.9406 | 1.0499 |
| CBS 13944 | 5.3481 | 0.1410 | 0.1375 | 20.2046 | 0.1528 |
| NBRC 0717 | 5.3998 | 0.1241 | -0.8090 | / | / |
| NBRC 1195 | 4.9776 | 0.1497 | 0.6267 | 4.4326 | 0.6965 |
| NBRC 1209 | 5.1831 | 0.1428 | 0.2410 | 11.5271 | 0.2678 |
| NBRC 1457 | 5.3672 | 0.1423 | 0.2111 | 13.1617 | 0.2346 |
| NBRC 1542 | 5.3614 | 0.1394 | 0.0509 | 54.6191 | 0.0565 |
| NBRC 1601 | 5.6142 | 0.1200 | -1.0435 | / | / |
| NBRC 1632 | 5.2753 | 0.1521 | 0.7643 | 3.6348 | 0.8494 |
| NBRC 1658 | 5.5030 | 0.1347 | -0.2123 | / | / |
| NBRC 1741 | 4.9623 | 0.1505 | 0.6706 | 4.1429 | 0.7452 |
| NBRC 1742 | 5.0269 | 0.1507 | 0.6825 | 4.0704 | 0.7585 |
| NBRC 10073 | 5.3940 | 0.1337 | -0.2732 | / | / |
| NRRL YB-271 | 5.0698 | 0.1576 | 1.0735 | 2.5879 | 1.1930 |
| NRRL YB-279 | 5.6712 | 0.1257 | -0.7196 | / | / |
| NRRL YB-387 | 5.7534 | 0.1208 | -0.9990 | / | / |
| NRRL YB-421 | 5.3045 | 0.1316 | -0.3886 | / | / |
| NRRL YB-423 | 4.9628 | 0.1448 | 0.3524 | 7.8835 | 0.3916 |
| NRRL YB-437 | 5.5275 | 0.1250 | -0.7596 | / | / |
| NRRL Y-323 | 5.3013 | 0.1587 | 1.1332 | 2.4516 | 1.2594 |
| NRRL Y-7149 | 5.2906 | 0.1573 | 1.0572 | 2.6279 | 1.1749 |
| NRRL Y-7317 | 5.0758 | 0.1511 | 0.7051 | 3.9398 | 0.7837 |
| NRRL Y-11853 | 5.7443 | 0.1391 | 0.0340 | 81.6173 | 0.0378 |
| NRRL Y-17622 | 5.3723 | 0.1492 | 0.5979 | 4.6465 | 0.6645 |
| NRRL Y-48157 | 5.4683 | 0.1272 | -0.6347 | / | / |
| NRRL Y-48651 | 5.8109 | 0.1388 | 0.0127 | 219.4286 | 0.0141 |
| NRRL Y-63530 | 5.4535 | 0.1532 | 0.8253 | 3.3663 | 0.9172 |
| EXF 11734 | 5.7853 | 0.1259 | -0.7100 | / | / |
| EXF 1446 | 5.5973 | 0.1366 | -0.1079 | / | / |
| EXF 6239 | 5.7223 | 0.1286 | -0.5563 | / | / |
| EXF 8413 | 6.0684 | 0.1280 | -0.5916 | / | / |
| EXF 8418 | 6.1973 | 0.1272 | -0.6349 | / | / |
| EXF 8861 | 5.8469 | 0.1243 | -0.7993 | / | / |
| EXF 9380 | 6.3348 | 0.1155 | -1.2955 | / | / |
| EXF 9502 | 6.3740 | 0.1147 | -1.3375 | / | / |
| EXF 9505 | 6.1851 | 0.1218 | -0.9402 | / | / |
| EXF 11732 | 5.7470 | 0.1299 | -0.4851 | / | / |
| EXF 11735 | 5.7665 | 0.1336 | -0.2760 | / | / |
| EXF 11879 | 5.9518 | 0.1387 | 0.0084 | 332.4874 | 0.0093 |
| EXF 12110 | 5.5097 | 0.1414 | 0.1599 | 17.3787 | 0.1777 |
| EXF 11947 | 5.5715 | 0.1304 | -0.4570 | / | / |
| W29-1 | 5.0524 | 0.1547 | 0.9110 | 3.0494 | 1.0125 |
| W29-2 | 5.0622 | 0.1543 | 0.8885 | 3.1266 | 0.9875 |
| Fos11 | 5.0122 | 0.1612 | 1.2745 | 2.1798 | 1.4164 |
| CLIB 80 | 5.3796 | 0.1409 | 0.1351 | 20.5697 | 0.1501 |
| CLIB 82 | 5.0841 | 0.1521 | 0.7628 | 3.6418 | 0.8478 |
| CLIB 83 | 5.2084 | 0.1418 | 0.1822 | 15.2478 | 0.2025 |
| CLIB 84 | 5.9565 | 0.1268 | -0.6613 | / | / |
| CLIB 86 | 6.6688 | 0.0989 | -2.2267 | / | / |
| CLIB 87 | 5.5383 | 0.1357 | -0.1609 | / | / |
| CLIB 205 | 5.2368 | 0.1426 | 0.2313 | 12.0098 | 0.2571 |
| CLIB 637 | 5.9366 | 0.1183 | -1.1367 | / | / |
| CLIB 632 | 5.8985 | 0.1163 | -1.2503 | / | / |
| CLIB 633 | 6.2312 | 0.1035 | -1.9700 | / | / |
| CLIB 634 | 5.8569 | 0.1231 | -0.8649 | / | / |
| CLIB 635 | 5.8337 | 0.1216 | -0.9534 | / | / |
| CLIB 713 | 5.9003 | 0.1234 | -0.8487 | / | / |
| CLIB 714 | 5.7142 | 0.1267 | -0.6628 | / | / |
| CLIB 715 | 5.6699 | 0.1327 | -0.3296 | / | / |
| CLIB 716 | 6.1581 | 0.1121 | -1.4853 | / | / |
| CLIB 717 | 5.8398 | 0.1202 | -1.0325 | / | / |
| CLIB 718 | 5.5638 | 0.1373 | -0.0711 | / | / |
| CLIB 721 | 4.7871 | 0.1600 | 1.2057 | 2.3041 | 1.3400 |
| CLIB 791 | 5.6506 | 0.1301 | -0.4740 | / | / |
| CLIB 879 | 5.9148 | 0.1182 | -1.1415 | / | / |
| CLIB 880 | 5.5970 | 0.1311 | -0.4146 | / | / |
| CLIB 881 | 5.5376 | 0.1348 | -0.2068 | / | / |
| CLIB 3040 | 5.5982 | 0.1311 | -0.4199 | / | / |
| CLIB 3073 | 5.4064 | 0.1340 | -0.2548 | / | / |
| CLIB 3088 | 5.4409 | 0.1204 | -1.0172 | / | / |
| ZIM 2116 | 5.1711 | 0.1527 | 0.7980 | 3.4812 | 0.8869 |
| ZIM 2409 | 5.3247 | 0.1350 | -0.1978 | / | / |
| ZIM 2413 | 5.0339 | 0.1441 | 0.3139 | 8.8496 | 0.3489 |
| ZIM 2416 | 5.3320 | 0.1337 | -0.2741 | / | / |
| ZIM 2423 | 5.0994 | 0.1323 | -0.3494 | / | / |
| ZIM 2439 | 5.6258 | 0.1269 | -0.6511 | / | / |
| ZIM 2449 | 5.1679 | 0.1254 | -0.7395 | / | / |
| ZIM 2450 | 5.2662 | 0.1419 | 0.1901 | 14.6119 | 0.2113 |
| ZIM 2511 | 5.6857 | 0.1231 | -0.8671 | / | / |
| ZIM 2584 | 5.9168 | 0.1323 | -0.3511 | / | / |
| NCAIM 00284 | 4.1209 | 0.2002 | 3.4678 | 0.8011 | 3.8539 |
| NCAIM 00759 | 5.5414 | 0.1323 | -0.3482 | / | / |
| NCAIM 00879 | 5.0390 | 0.1621 | 1.3258 | 2.0954 | 1.4734 |
| NCAIM 00587 | 5.3440 | 0.1319 | -0.3717 | / | / |
| NCAIM 01087 | 5.0076 | 0.1505 | 0.6722 | 4.1326 | 0.7471 |
| DBVPG 3070 | 5.7444 | 0.1096 | -1.6255 | / | / |
| DBVPG 3219 | 5.7427 | 0.1167 | -1.2256 | / | / |
| DBVPG 3220 | 5.2503 | 0.1222 | -0.9166 | / | / |
| DBVPG 3374 | 6.3166 | 0.1132 | -1.4226 | / | / |
| DBVPG 3375 | 4.5524 | 0.1732 | 1.9496 | 1.4249 | 2.1667 |
| DBVPG 3376 | 4.8752 | 0.1718 | 1.8684 | 1.4869 | 2.0764 |
| DBVPG 3536 | 4.4066 | 0.1767 | 2.1448 | 1.2952 | 2.3837 |
| DBVPG 4399 | 5.0691 | 0.1583 | 1.1106 | 2.5014 | 1.2343 |
| DBVPG 4400 | 4.4991 | 0.1725 | 1.9088 | 1.4554 | 2.1214 |
| DBVPG 4401 | 5.2514 | 0.1454 | 0.3875 | 7.1691 | 0.4307 |
| DBVPG 4556 | 5.1690 | 0.1476 | 0.5079 | 5.4695 | 0.5645 |
| DBVPG 4557 | 5.1645 | 0.1404 | 0.1074 | 25.8663 | 0.1194 |
| DBVPG 4558 | 5.4435 | 0.1310 | -0.4212 | / | / |
| DBVPG 5851 | 4.4804 | 0.1880 | 2.7781 | 1.0000 | 3.0875 |
| DBVPG 5855 | 5.0881 | 0.1453 | 0.3830 | 7.2533 | 0.4257 |
| DBVPG 5858 | 5.1193 | 0.1545 | 0.8987 | 3.0913 | 0.9987 |
| DBVPG 6736 | 4.5493 | 0.1752 | 2.0615 | 1.3476 | 2.2911 |
| DBVPG 6737 | 5.6095 | 0.1377 | -0.0471 | / | / |
| DBVPG 6868 | 5.9238 | 0.1378 | -0.0415 | / | / |
| DBVPG 7042 | 4.8778 | 0.1535 | 0.8407 | 3.3046 | 0.9343 |
| NRRL Y-7320 | 5.5963 | 0.1303 | -0.4633 | / | / |
| NRRL Y-7754 | 5.6213 | 0.1390 | 0.0284 | 97.7743 | 0.0316 |
| NRRL Y-11854 | 4.8114 | 0.1627 | 1.3593 | 2.0438 | 1.5106 |
| NRRL Y-63532 | 5.2571 | 0.1485 | 0.5603 | 4.9581 | 0.6227 |
| NRRL Y-5383 | 5.4540 | 0.1375 | -0.0585 | / | / |
| NRRL YB-272 | 6.4117 | 0.1256 | -0.7255 | / | / |
| NRRL YB-276 | 4.5483 | 0.1782 | 2.2301 | 1.2457 | 2.4784 |
| NRRL YB-281 | 5.3617 | 0.1538 | 0.8582 | 3.2371 | 0.9538 |
| NRRL YB-419 | 4.8201 | 0.1584 | 1.1167 | 2.4878 | 1.2411 |
| NRRL YB-420 | 6.8949 | 0.1098 | -1.6143 | / | / |
| NRRL YB-566 | 5.5426 | 0.1339 | -0.2594 | / | / |
| NRRL Y-7318 | 5.2726 | 0.1452 | 0.3741 | 7.4269 | 0.4157 |
| NRRL Y-48653 | 5.5107 | 0.1284 | -0.5674 | / | / |
| NRRL Y-1095 | 5.2326 | 0.1385 | -0.0025 | / | / |
| NRRL Y-7208 | 5.8178 | 0.1278 | -0.6023 | / | / |
| NRRL Y-17534 | 5.2697 | 0.1447 | 0.3476 | 7.9926 | 0.3863 |
| NRRL Y-63744 | 4.9068 | 0.1605 | 1.2331 | 2.2530 | 1.3704 |
| NRRL YB-392 | 5.5028 | 0.1487 | 0.5722 | 4.8553 | 0.6359 |
| NRRL YB-418 | 5.5831 | 0.1300 | -0.4815 | / | / |
| JCM 2318 | 4.9169 | 0.1548 | 0.9136 | 3.0407 | 1.0154 |
| JCM 2338 | 5.1259 | 0.1457 | 0.4026 | 6.8999 | 0.4475 |
| JCM 8061 | 5.1703 | 0.1472 | 0.4855 | 5.7220 | 0.5396 |
| JCM 21924 | 4.7098 | 0.1491 | 0.5937 | 4.6790 | 0.6599 |
| PYCC 4454 | 4.9670 | 0.1464 | 0.4449 | 6.2436 | 0.4945 |
| PYCC 4743 | 5.3016 | 0.1409 | 0.1344 | 20.6740 | 0.1493 |
| PYCC 4811 | 5.2742 | 0.1535 | 0.8386 | 3.3128 | 0.9320 |
| PYCC 4936 | 4.9726 | 0.1585 | 1.1197 | 2.4810 | 1.2444 |
| PYCC 5201 | 4.2895 | 0.1914 | 2.9717 | 0.9349 | 3.3026 |
| CICC 1440 | 4.6002 | 0.1754 | 2.0723 | 1.3406 | 2.3031 |
| CICC1444 | 4.7440 | 0.1743 | 2.0112 | 1.3813 | 2.2351 |
| CICC 31577 | 4.8532 | 0.1553 | 0.9440 | 2.9430 | 1.0491 |
| CICC 31588 | 5.6722 | 0.1200 | -1.0406 | / | / |
| CICC 31596 | 4.6780 | 0.1631 | 1.3819 | 2.0103 | 1.5358 |
| CICC 32859 | 4.9989 | 0.1635 | 1.4009 | 1.9831 | 1.5569 |
| TBRC 15324 | 5.0025 | 0.1475 | 0.5051 | 5.5004 | 0.5613 |
| TBRC 1747 | 5.1173 | 0.1463 | 0.4386 | 6.3334 | 0.4875 |
| TBRC 3999 | 5.2234 | 0.1370 | -0.0878 | / | / |
| TBRC 4320 | 5.1201 | 0.1380 | -0.0297 | / | / |
| TBRC 4337 | 4.9551 | 0.1420 | 0.1951 | 14.2368 | 0.2169 |
| TBRC 4346 | 5.0930 | 0.1407 | 0.1236 | 22.4775 | 0.1374 |
| TBRC 4382 | 5.1305 | 0.1456 | 0.3968 | 7.0008 | 0.4410 |
| TBRC 4416 | 5.0404 | 0.1393 | 0.0453 | 61.2627 | 0.0504 |
| TBRC 4417 | 5.3469 | 0.1349 | -0.2048 | / | / |
| TBRC 4421 | 5.3799 | 0.1279 | -0.5992 | / | / |
| TBRC 4434 | 5.3534 | 0.1271 | -0.6408 | / | / |
| CGMCC2.1207 | 5.1378 | 0.1418 | 0.1854 | 14.9851 | 0.2060 |
| CGMCC2.1216 | 5.3548 | 0.1422 | 0.2058 | 13.4963 | 0.2288 |
| CGMCC2.1383 | 4.8197 | 0.1497 | 0.6271 | 4.4301 | 0.6969 |
| CGMCC2.1712 | 5.3229 | 0.1390 | 0.0257 | 108.0923 | 0.0286 |
| CGMCC2.1713 | 4.8691 | 0.1538 | 0.8575 | 3.2396 | 0.9530 |
| CGMCC2.1715 | 4.8113 | 0.1378 | -0.0406 | / | / |
| CGMCC2.1718 | 6.2159 | 0.1194 | -1.0729 | / | / |

* *OD*_600 (increased)_= *OD*_600_ at the end - initial *OD*_600_ (0.2).

**Table S6** **Comparison of homology arm sequence on p4906-ku70-Cas9 with a sequence on the corresponding locus of DBVPG 5851**

| Sequences producing significant alignments | | Score | E-value |
| --- | --- | --- | --- |
| contig00008 | len=379559 | 10385 | 0 |
| contig00002 | len=604935 | 48.2 | 4.00E-04 |
| contig00152 | len=29339 | 43.7 | 0.016 |
| contig00121 | len=48745 | 43.7 | 0.016 |
| contig00086 | len=75872 | 41 | 0.054 |
| contig00044 | len=152070 | 41 | 0.054 |
| contig00079 | len=83889 | 40.1 | 0.19 |
| contig00168 | len=23123 | 39.2 | 0.19 |
| contig00067 | len=105467 | 39.2 | 0.19 |
| contig00026 | len=199315 | 39.2 | 0.19 |
| Result >>> contig00008: Score=10385 bits, Expect=0.0, Identities=5758/5758 (100%), Gaps=0/5758 (0%) | | | |

**Table S7 The sequences of the various signal peptides used in this study and their related information**

| Signal peptides | Encoded protein | Amino acid sequence | Nucleotide sequence (5’→3’) | Source |
| --- | --- | --- | --- | --- |
| XPR2 pre | P09230 | MKLATAFTIL TAVLA | ATGAAGCTCGCTACCGCCTTTACTATT CTCACTGCCGTTCTGGCC | Literature |
| *YALI0B03564g* | Q6CFU7 | MKFTFAAVTA ALASSAMA | ATGAAGTTCACATTTGCTGCCGTTACC GCCGCGCTGGCCTCGTCCGCCATGGCC | Secretome |
| *YALI0D20680g* | Q6C8C9 | MKFSTALLAL AAVATA | ATGAAGTTCTCCACCGCCCTTCTGGCT CTGGCCGCCGTCGCCACCGCC | Secretome |
| *YALI0E07744p* | Q6C6P1 | MHFSFGLLLA ASSVLA | ATGCACTTTTCGTTTGGCCTTCTGCTG GCCGCCTCTTCGGTTCTGGCT | Secretome |

**Table S8 Relative activity of extracellular recombinant rPPase**

| Strain Name | Integrated signal peptide | Enzyme activity of rPPase (U/mL) |
| --- | --- | --- |
| YYL 2575 | XPR2 pre | 2.131±0.255 |
| YYL 2576 | *YALI0B03564g* | 0.866±0.121 |
| YYL 2577 | *YALI0D20680g* | 5.537±0.159 |
| YYL 2578 | *YALI0E07744p* | 4.953±0.438 |

**Supporting Figures & Figure Captions**

**
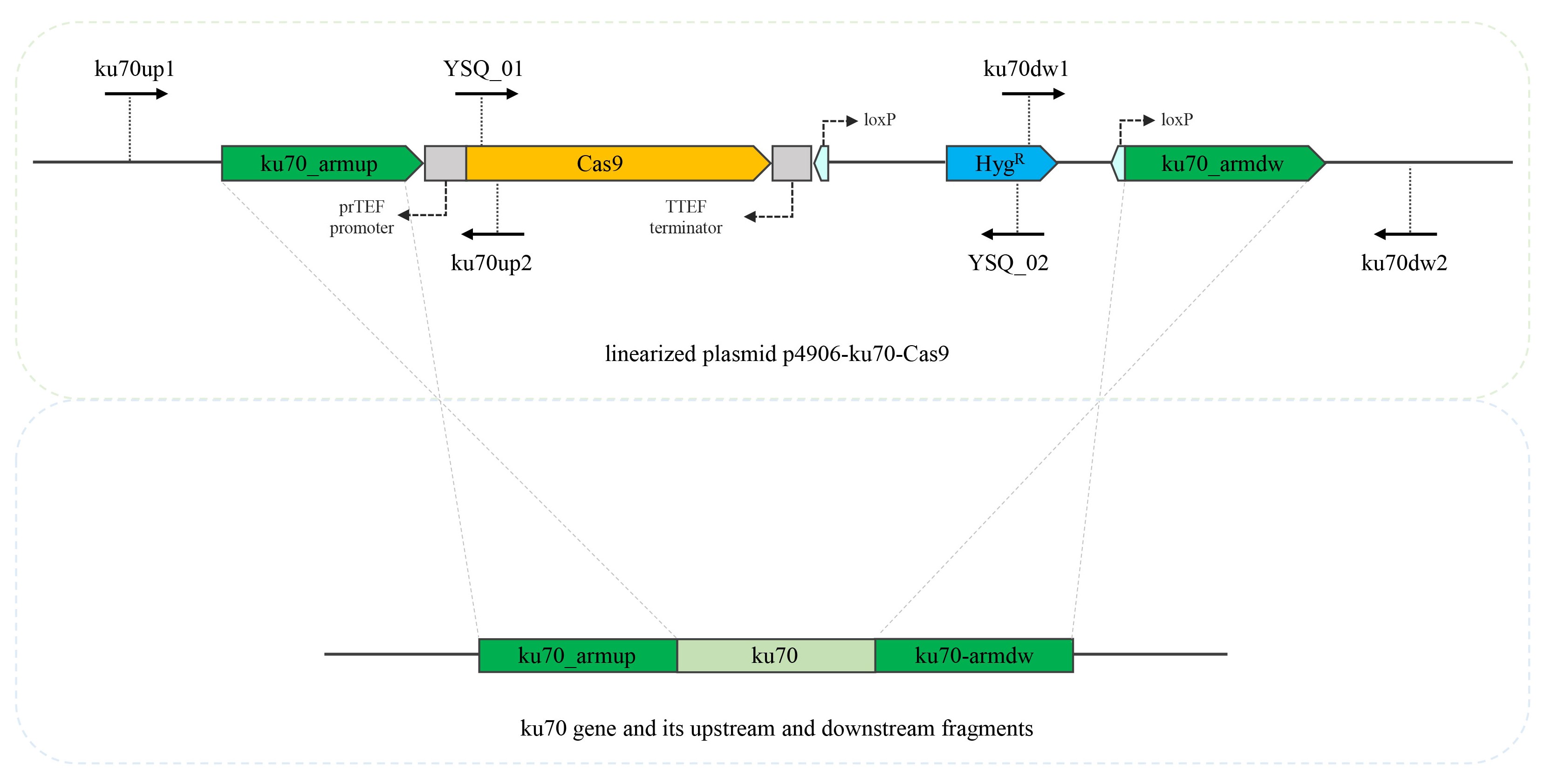
**

**Fig. S1 Verification of PCR-based knockout of the *ku70* and knock-in of the gene encoding the Cas9.**


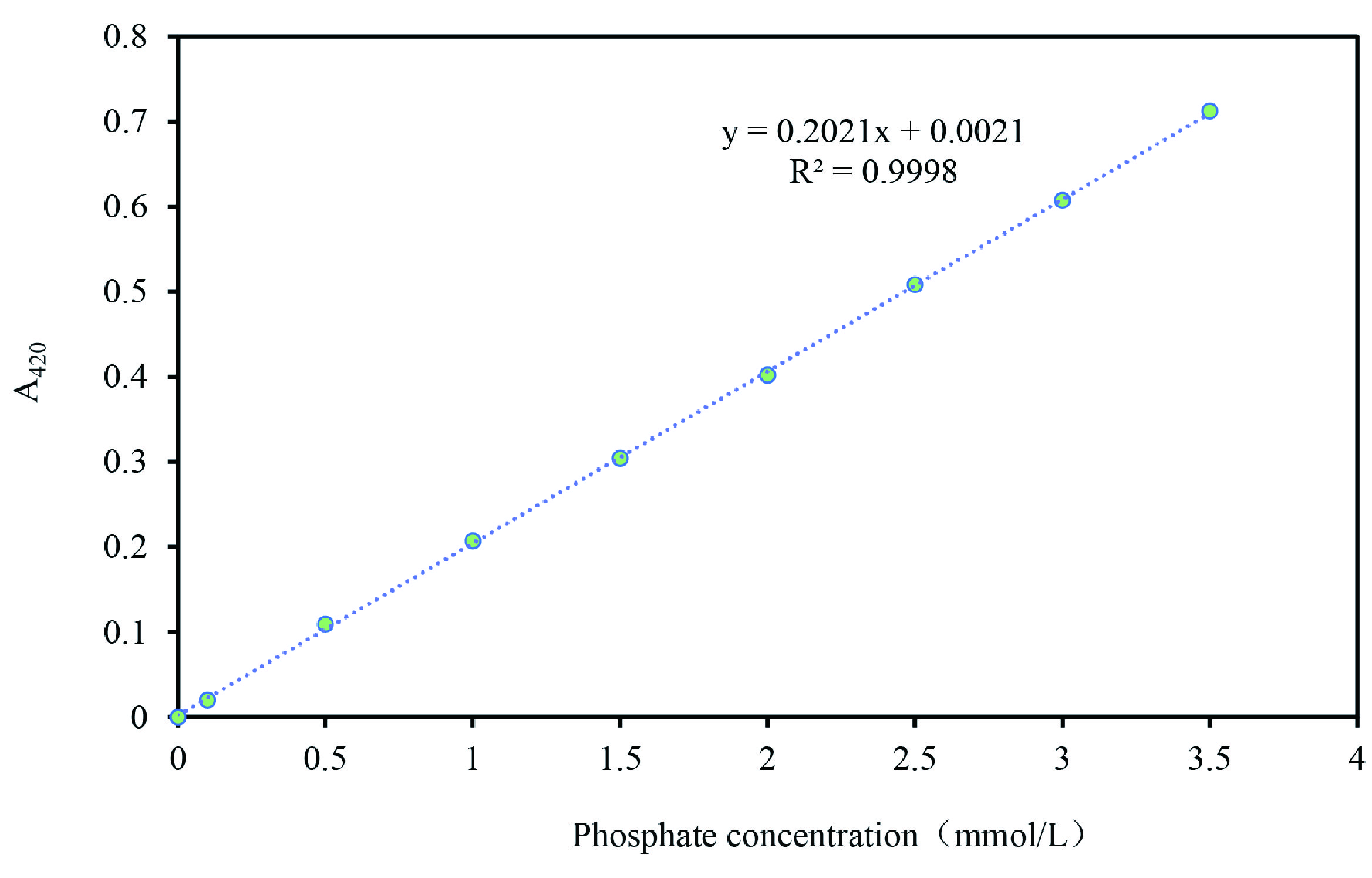


**Fig. S2 Establishment of the standard curve for detecting phosphate.**
